# Impaired AMPARs Translocation into Dendritic Spines with Motor Skill Learning in the Fragile X Mouse Model

**DOI:** 10.1523/ENEURO.0364-22.2023

**Published:** 2023-03-24

**Authors:** Anand Suresh, Anna Dunaevsky

**Affiliations:** 1 Department of Biochemistry and Molecular Biology, University of Nebraska Medical Center, Omaha NE 68198; 2 Department of Neurological Sciences, University of Nebraska Medical Center, Omaha, NE 68198

**Keywords:** AMPAR, dendritic spine, FMRP, learning, motor cortex

## Abstract

Motor skill learning induces changes in synaptic structure and function in the primary motor cortex (M1). In the fragile X syndrome (FXS) mouse model an impairment in motor skill learning and associated formation of new dendritic spines was previously reported. However, whether modulation of synaptic strength through trafficking of AMPA receptors (AMPARs) with motor skill training is impaired in FXS is not known. Here, we performed *in vivo* imaging of a tagged AMPA receptor subunit, GluA2, in layer (L)2/3 neurons in the primary motor cortex of wild-type (WT) and *Fmr1* knock-out (KO) male mice at different stages of learning a single forelimb-reaching task. Surprisingly, in the *Fmr1* KO mice, despite impairments in learning there was no deficit in motor skill training-induced spine formation. However, the gradual accumulation of GluA2 in WT stable spines, which persists after training is completed and past the phase of spine number normalization, is absent in the *Fmr1* KO mouse. These results demonstrate that motor skill learning not only reorganizes circuits through formation of new synapses, but also strengthens existing synapses through accumulation of AMPA receptors and GluA2 changes are better associated with learning than new spine formation.

## Significance Statement

This study identifies a significant synaptic defect associated with a behavioral impairment relevant to the pathology of fragile X syndrome (FXS). Using *in vivo* imaging of a tagged AMPA-type receptor subunit GluA2, we found that the motor-skill training-induced accumulation of GluA2 in dendritic spines that occurs in control mice is impaired in the *Fmr1* knock-out (KO) mouse. This study identifies a synaptic correlate of impaired motor skill learning in the *Fmr1* KO mouse.

## Introduction

In the mammalian central nervous system, AMPA-type glutamate receptors mediate the vast majority of fast excitatory synaptic transmission. Synaptic strengthening through mechanisms of long-term potentiation (LTP) involves insertion of AMPA receptors (AMPARs) into synapses and is believed to be critical for learning and memory (Malinow and Malenka, 2002; Takahashi et al., 2003; Matsuzaki et al., 2004; Luscher and Malenka, 2012). Previous studies have demonstrated that with motor skill learning new dendritic spines, sites of excitatory synaptic input, are formed and stabilized in the primary motor cortex (M1; Xu et al., 2009; Yang et al., 2009; Padmashri et al., 2013; Reiner and Dunaevsky, 2015). Moreover, slice electrophysiology experiments demonstrated strengthening of cortical connections in M1 with motor learning (Rioult-Pedotti et al., 1998; Harms et al., 2008) and spines were both larger (Harms et al., 2008; Hayashi-Takagi et al., 2015) and accumulated the GluA1 subunit of AMPAR with learning (Padmashri et al., 2013; Roth et al., 2020). Both motor skill learning and structural and functional synaptic plasticity induced by motor skill training are impaired in the *Fmr1* knock-out (KO) mouse, a model for fragile X syndrome (FXS; Padmashri et al., 2013; Reiner and Dunaevsky, 2015). It was previously shown that the GluA2 subunit of AMPAR is highly dynamic in dendritic spines *in vivo* and that their dynamics are altered in the *Fmr1* KO mouse (Suresh and Dunaevsky, 2017), but if learning induced translocation of AMPAR is impaired in FXS is not known. In this study, we set out to understand whether motor skill learning modulates GluA2 in dendritic spines and whether it is impaired in the FXS mouse model.

Here, we bilaterally electroporated (Saito and Nakatsuji, 2001) L2/3 pyramidal neurons in the M1 of *Fmr1* KO and wild-type (WT) mice with the AMPAR GluA2 subunit tagged with a pH-sensitive form of GFP (Super Ecliptic pHluorin, sGluA2; Miesenbock et al., 1998) leading to overexpression of GluA2 and td-Tomato as a morphologic marker. We then monitored dynamics of AMPARs and dendritic spines in anesthetized animals through a cranial window with two-photon microscopy (Holtmaat et al., 2009; Suresh and Dunaevsky, 2017) during 5 d of a single-pellet forelimb-reaching task. To determine the long-term effects of training on synaptic GluA2, we further imaged the same spines for up to 10 d. We found that there was a transient increase in the number of dendritic spines in the hemisphere contralateral to the trained forelimb of both WT and KO mice. Importantly, we found that sGluA2 accumulated in spines in the contralateral hemisphere to the trained forelimb of WT but not *Fmr1* KO mice. These results suggest that in addition to restructuring of the circuit through formation and stabilization of new spines, motor skill learning results in strengthening of preexisting synapses which is impaired in FXS.

## Materials and Methods

### Experimental model and subject details

Mice were kept on a regular light/dark cycle. Female C57BL/6 *Fmr1* heterozygous (HET) mice were crossed with male C57BL/6 *Fmr1* KO mice and used for *in utero* electroporation (a total of 10 litters). Since FXS predominantly affects males, male WT littermates and *Fmr1* KO pups were used for all experiments. Mice were cared for in accordance with National Institutes of Health *Guidelines for Laboratory Animal Welfare*. All experiments were approved by the Institutional Animal Care and Use Committee.

### Method details

#### Motor skill training

Training was performed as previously described (Padmashri et al., 2013). Seven-week-old mice following cranial window implantation were food-restricted (85% of their free-feeding weight). Food was removed the night before the first training session. The WT and KO mice did not differ in their weight after the first day of food restriction (WT: 16.23 ± 0.65 g; KO: 17.34 ± 0.73 g, *p* = 0.29). During training, mice were fed food pellets (Dustless Precision Pellets 20 mg, rodent grain-based diet, Bioserv catalog #F0163) during and after the training session. All training was done in the afternoon to reduce effects of circadian variations on training. Before food restriction, mice were habituated for half an hour in a Plexiglas box with an attached platform that could be reached through a thin slit on the front wall. Mice were trained to reach through the slit with their preferred forelimb and grasp and retrieve individual food pellets. An initial pretraining session determined forelimb preference, and pellets were then placed on the side that enabled the use of the preferred forelimb only. Mice had one training session per day that lasted 30 min or 100 reaches. Motor skill performance was quantified by the success rate (% of successful retrievals) and was normalized to day 1. The contralateral and ipsilateral hemispheres to the trained forelimb are abbreviated as CH and IH, respectively. All trained mice were used for imaging however, in some mice only a single hemisphere was imaged.

#### DNA constructs

We used FUGW pUB-SEP-GluA2-WPRE and pCAG-tdTom constructs for our experiments. FUGW pUB-SEP-GluA2-WPRE was a generous gift from the lab of Noam Ziv (Zeidan and Ziv, 2012). For the morphologic marker, we excised DsRed2 from addgene Plasmid #11151 using the KpnI and NotI restriction sites and cloned tdTomato into the vector under the CAG promoter using In-Fusion HD Cloning kits (Clonetech Takara Bio, catalog #639649).

#### Surgical procedures

##### *In utero* electroporation

**Figure 1.**
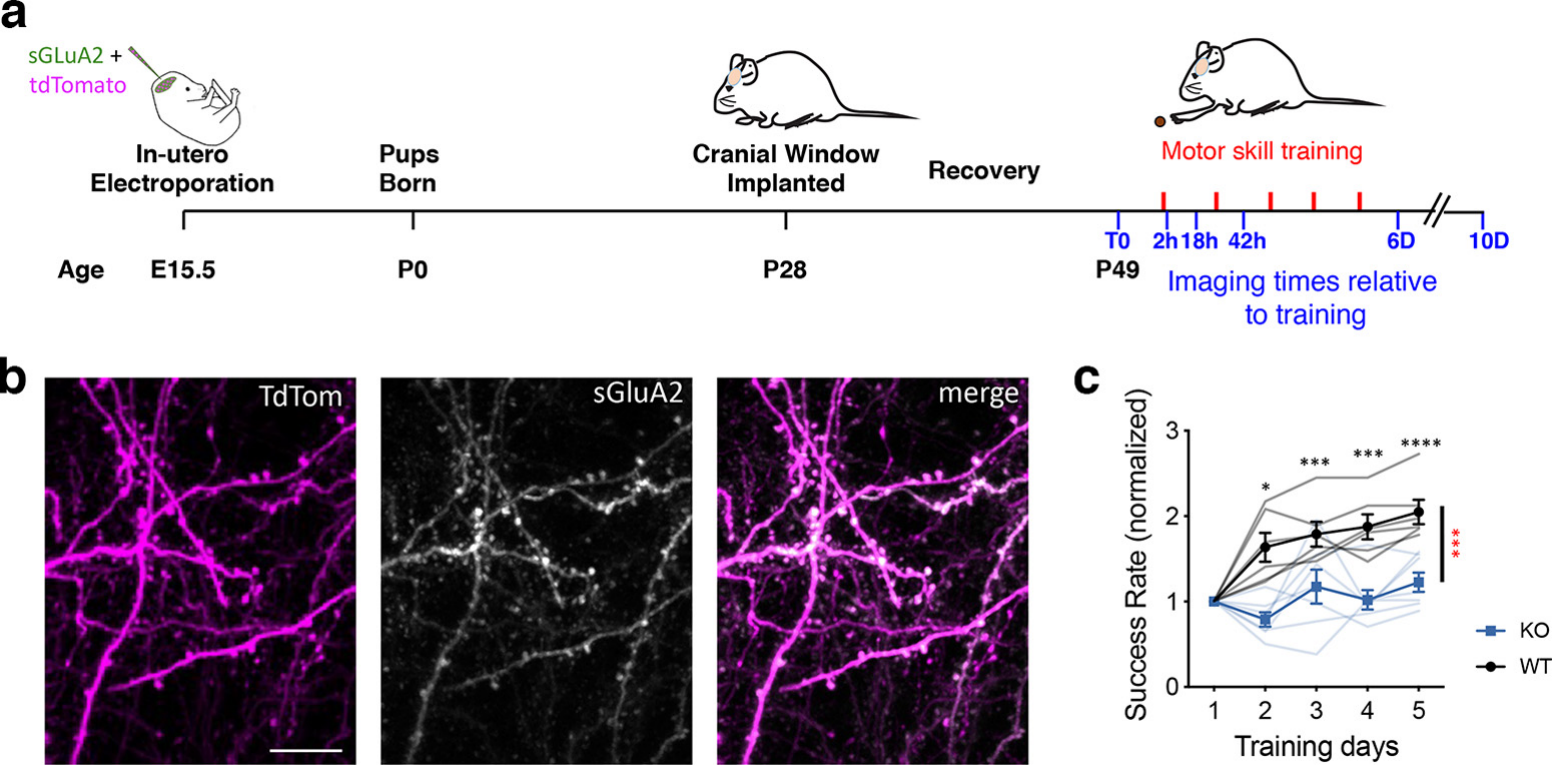
*In vivo* imaging of AMPAR with motor skill training. ***a***, Schematic of the experimental design. ***b***, Images of transfected region of cortex showing overlap of tdTom (magenta) and sGluA2 (white). Scale bar = 15 μm. ***c***, Behavioral performance (normalized success rate) of mice trained on forelimb reaching task. Thin lines represent individual mice and the bold line is the average [*Fmr1* KO, blue, *n* = 6 mice and litter mate control (WT) black, *n* = 7 mice] mean ± SEM. Two-way repeated measures ANOVA followed by one-way ANOVA with Bonferroni correction. Genotype × Time, *F*_(4,44)_ = 7.03, *p* = 0.0002, *p < 0.05, ***p < 0.001, and ****p < 0.0001. Red stars indicate WT versus KO comparisons, and black stars indicate D1 versus D_i_. See Extended Data Figure 1-1 and Extended Data Table 1-1 for more information.

Timed pregnant female C57BL/6 *Fmr1* HET mice were *in utero* electroporated as described previously (Fig. 1*a*; Suresh and Dunaevsky, 2017). Briefly, embryonic day (E)15.5 timed pregnant C57BL/6 *Fmr1* HET mice were injected with Buprenorphine (0.1 mg/kg) 30 min before the surgery. Following this, the dams were anaesthetized using an isoflurane-oxygen mixture (induction: 5% isoflurane/2 l/min O_2_, maintenance: 2% isoflurane/2 l/min O_2_). A small incision was made within the abdominal walls and uterine horns were exposed. 0.5 μl of a 4 μg/μl DNA solution of pCAG-tdTomato and pUB-SEP-GluA2-WPRE was injected into the cerebral lateral ventricles of E15.5 mouse embryo using a pulled glass pipette (BF100-94 Sutter Instrument) and Parker Picospritzer III microinjection system. The head was then placed between 3-mm tweezer electrodes so as to target the motor cortex. Electroporation was achieved using five square pulses (5 ms long at 1 Hz, 35 mV) using BTX Harvard Electro Square Porator ECM 830. Embryos were returned back into the abdominal cavity, and the abdominal muscles were sutured using non absorbable sutures (Ethicon Permahand). The dams were revived and monitored for distress over a period of 24 h after surgery. Dams were allowed to deliver naturally.

10.1523/ENEURO.0364-22.2023.f1-1 Extended Data Figure 1-1 Motor skill learning and number of attempts. ***a***, Behavioral performance (success rate) of mice trained on forelimb reaching task. Thin lines represent individual mice and the bold line is the average [*Fmr1* KO, blue, *n* = 6 mice and litter mate control (WT) black, *n* = 7 mice] mean ± SEM. Two-way repeated measures ANOVA followed by one-way ANOVA with Bonferroni correction. Genotype × Time, *F*_(4,44)_ = 6.07, *p* = 0.0006. ***b***, Number of attempts performed. Genotype × Time, *F*_(4,44)_ =1.916, *p* = 0.12 Download Figure 1-1, TIF file.

10.1523/ENEURO.0364-22.2023.tab1-1 Extended Data Table 1-1 Full statistical information for Figure 1. Download Table 1-1, DOCX file.

##### Cranial window

At postnatal day (P)28–P30, mice were anesthetized with tribromoethanol (Avertin, 0.25 mg/g body weight) and a cranial window was implanted over the motor cortex (Fig. 1*a*). Briefly, half an hour before the surgery, dexamethasone (∼2 μg/g body weight) and carprofen (5 μg/g body weight) were injected intraperitoneally to reduce cerebral edema and inflammation during the craniotomy. A 5-mm craniotomy centered on bregma, was made across the sutures, above the primary motor cortex. After the craniotomy, the exposed surgery site was rinsed with an enrofloxacin antibiotic solution (0.5 μg/ml) and covered with a 5-mm-diameter cover glass, which was permanently glued to the skull using dental acrylic cement. The dura remained intact in this procedure. Mice were treated with antibiotic enrofloxacin (5 mg/kg) twice daily for 6 d after surgery to prevent bacterial infection. Mice were also injected daily with carprofen (5 mg/kg) for three weeks following surgery to reduce inflammation. Mice were allowed three weeks to recover from the surgery.

#### Imaging and image analysis

##### Two-photon imaging

All imaging was performed with a multiphoton microscope (Moving Objective Microscope, MOM; Sutter), using a Ti:Sapphire laser (Chameleon Vision II, Coherent) tuned to 925 nm. Imaging time points (2 h, 18 h, 42 h, 6 d, and 10 d after first day of training) were chosen to capture early changes in spines and GluA based on our previous study (Padmashri et al., 2013). Mice were anaesthetized with a ketamine/dexdormitor mixture (100 and 0.5 mg/ml, respectively, 2.5 ml/kg). Images were collected with a Nikon water-immersion objectives (25×, 1.05 NA). Excitation power measured at the back aperture of the objective was typically ∼20 mW and was adjusted to achieve near identical levels of fluorescence for each imaged region using a Pockels cell. Two-channel imaging was achieved by using a 670-nm dichroic mirror and two external photomultiplier tubes. A 535/50 bandpass filter was used to detect sGluA2 emission and a 610/75 bandpass filter was used to detect tdTomato. For imaging, we used ScanImage software written in MATLAB (MathWorks; Pologruto et al., 2003). During an imaging session, six to ten regions of interest (ROIs) per animal were selected along the dendritic tufts of tdTomato-expressing and sGluA2-expressing layer 2/3 pyramidal neurons. All imaged dendrites were in layer 1 (within the first 100 μm below the dura matter) within the forelimb M1, as determined by stereotaxic measurements (between 750 and 2000 μm lateral to the midline and between 1000 μm rostral and 250 μm caudal from bregma; Tennant et al., 2011). Each ROI consisted of a stack of images (20–80 optical sections, separated axially by 1 μm). The coordinates of each ROI were recorded using the XYZ motor on the MOM for subsequent imaging days. After imaging, mice were revived from anesthesia with Antisedan (atipamezole hydrochloride 5.0 mg/ml).

##### Spine identification

**Figure 2.**
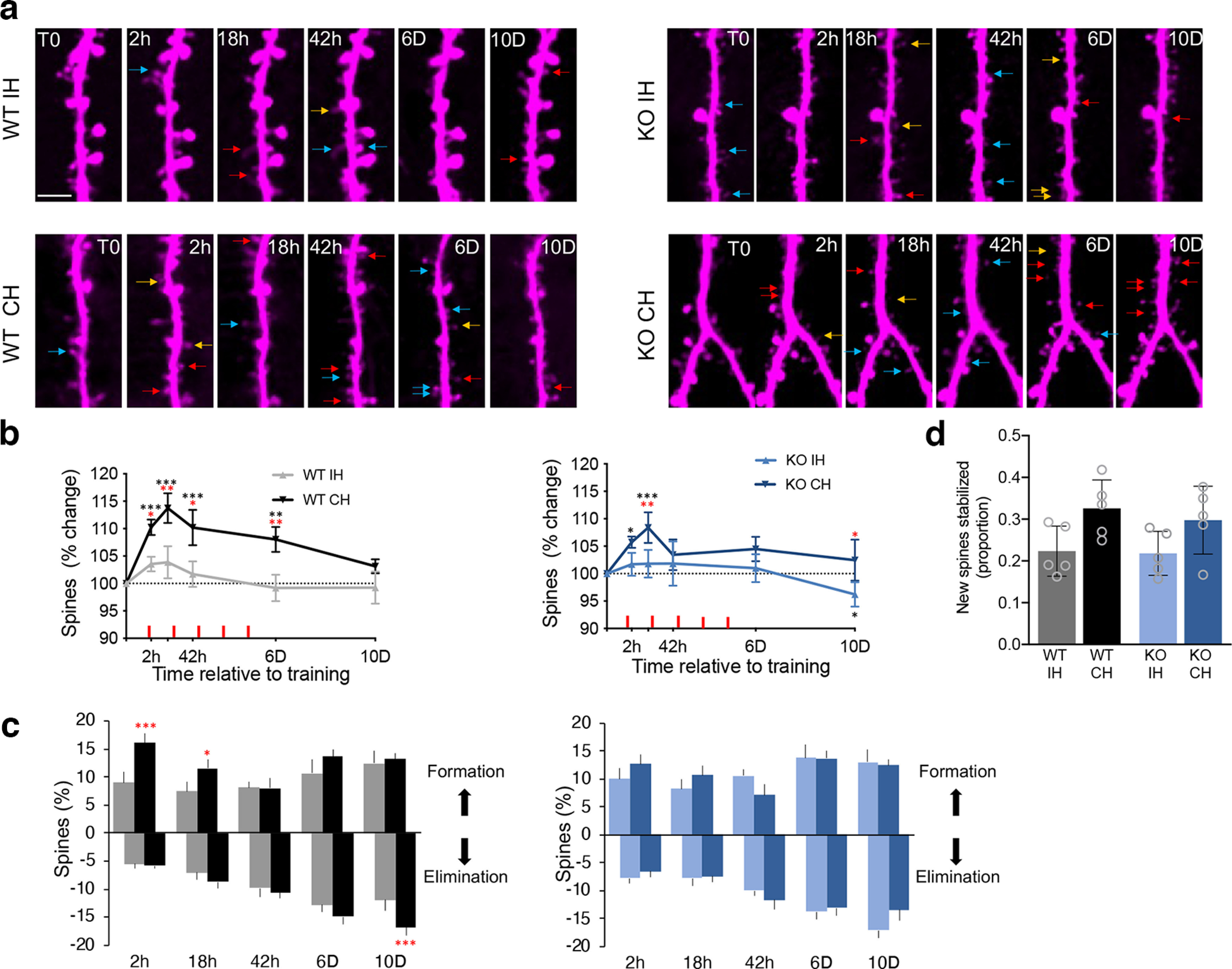
Motor skill training results in transient increase in spines in WT and *Fmr1* KO mice. ***a***, Images of layer 2/3 neuron dendrites in the forelimb M1 of WT and *Fmr1* KO mice imaged before (T0) and at different times after the first training session. The hemispheres ipsilateral and contralateral to the trained forelimb are referred to IH and CH, respectively. Red, blue, and yellow arrows mark newly formed spines, spines eliminated in the following imaging session, and new spines that were eliminated in the following imaging session, respectively. Scale bar = 10 μm. ***b***, Total spine number. WT-IH (gray) 36 dendrites, 881 spines at T0. WT-CH (black) 37 dendrites, 963 spines at T0. KO-IH (light blue) 35 dendrites, 911 spines at T0. KO-CH (dark blue) 36 dendrites, 965 spines at T0. *n* = 5 mice per group. Nested random effects mixed model analysis. Genotype, *F*_(1,9.5)_ = 0.72, *p* = 0.42; Training, *F*_(1,61.4)_ = 21.25, *p* < 0.0001; Time, *F*_(4,564. 9)_ = 12.99, *p* < 0.0001. Red stars indicate CH versus IH comparisons and black stars indicate T0hr versus T_i_. *p < 0.05, **p < 0.01, and ***p < 0.001. ***c***, Spine formation and elimination between imaging days. Nested random effects mixed model analysis. Formation: Genotype × Hemisphere, *F*_(1,86.84)_ = 6.98, *p* < 0.01; Genotype × Time, *F*_(4,560)_ = 1.45, *p* = 0.22; Hemisphere × Time, *F*_(4,560)_ = 5.77, *p* = 0.0001. Elimination: Genotype × Hemisphere, *F*_(1,100.3)_ = 6.06, *p* = 0.016; Time, *F*_(1,563.2)_ = 44.43, *p* < 0.0001; Genotype × Time, *F*_(1,563.2)_ = 0.58, *p* = 0.68; Hemisphere × Time, *F*_(1,563.2)_ = 0.46, *p* = 0.76; Genotype × Hemisphere × Time, *F*_(1,563.2)_ = 2.19, *p* = 0.07. Stars indicate IH versus CH. mean ± SEM *p < 0.05 and ***p < 0.001. ***d***, Proportion of new spines (formed at 2 or 18 h after training) that were stable until the last imaging day. WT-IH (135) spines, WT-CH (264 spines), KO-IH (178 spines), KO-CH (226 spines), mean ± SEM, *n* = 5 mice per group. Two-way ANOVA with Sidak correction. Hemisphere, *F*_(1,16)_ = 9.39, *p* = 0.007; Hemisphere × Genotype, *F*_(1,16)_ = 0.15, *p* = 0.7. See Extended Data Table 2-1 for more information.

Images were corrected for tdTomato bleed-through into sGluA2 (green) channel by quantifying percent bleed-through on a dTomato only expressing mouse and subsequently subtracting out the bleed-through from images of the sGluA2 (green) channel images (bleed-through was minimal <0.05%). A custom written imaging program written in Python was used to track dendritic spines and sGluA2 levels over imaging sessions. Dendritic segments of 30–80 μm were chosen in three-dimensional stacks and dendritic spines were identified and traced in the dTomato image channel on images from the first imaging session (T0). A spine had to be >0.5 μm (four pixels) perpendicular from the shaft of the dendrite to be considered a spine. Unless mentioned, T0 images were considered as baseline for all analysis. For each dendritic segment, % change in spines was calculated and averages per mouse per condition are presented (Fig. 2*b*). For spine dynamic analysis, images were compared with images from previous time points (except for baseline images) and categorized as stable if they were present in both images, eliminated if they appeared in the previous image but not in the image being analyzed, and newly formed when they appeared in the image being analyzed but not in the baseline image. Spine formation and elimination was calculated as a percentage of new or eliminated spines of the total number of spines at the imaging period (Holtmaat et al., 2005).

10.1523/ENEURO.0364-22.2023.f2-1 Extended Data Figure 2-1 Increased basal level spine density in the *Fmr1* KO mouse. Download Figure 2-1, TIF file.

10.1523/ENEURO.0364-22.2023.tab2-1 Extended Data Table 2-1 Full statistical information for Figure 2. Download Table 2-1, DOCX file.

##### sGluA2 and spine intensity measurement

Images were background corrected and normalized across imaging sessions using the dendrite value as previously described (Suresh and Dunaevsky, 2017). To quantify sGluA2 and spine intensity, spines were identified using the tdTomato channel across all time points. Regions of interest were traced along the boundaries of the spines using the brightest frame of the tdTomato channel as reference. Once an ROI was traced, sGluA2 signal was calculated within the ROI as the sum of total integrated pixel intensity across the three brightest optical frames of the spine. These measurements were calculated for both tdTomato and sGluA2 channel and individually corrected for background. To normalize across imaging sessions, first, imaging conditions were kept constant, and second, the tdTomato intensity of the dendrites was used to normalize sGluA2 and spine signal. Two squares (four pixels by four pixels) were traced along the dendrite adjacent to the spine and the average of the brightest frames of the dendrite ROIs was used to normalize the spine values. Normalization, was performed individually for each spine. At each time point the corrected and normalized spine value was normalized to the baseline imaging session (T0), resulting in fold-change values. We present the geometrical means as to not over represent increases (1 to infinity) versus decreases (0–1).

For presentation purposes, all images were de-speckled and smoothened using median filters in ImageJ. Traversing axons were removed, and three to six frames were maximally projected. All analysis was done blinded to mouse genotype on unprocessed images except for the bleed-through correction described above.

##### Percentile spine grouping

sGluA2 intensity for all spines within a dendrite (30–80 μm) at baseline were arranged in ascending order and percentile rank for every spine was calculated. Spines were divided into four percentile groups (bin width of 25%) with progressively increasing levels of sGluA2. Within each mouse the sGluA2 percentage changes at each time point were quantified. However, because smaller spines are less stable than large spines, the number of persistent spines is not identical in each of the four groups (Extended Data Fig. 3-5). Unless indicated otherwise, the averages were calculated per mouse.

##### sGluA2 spine dynamic grouping

sGluA2 dynamics were quantified as percentage change of sGluA2 at all time points compared with the previous time point. We defined change as ±2 SD of the percentage change at 24 h in WT, which sets a threshold of ±30% (Zhang et al., 2015; Suresh and Dunaevsky, 2017). Spines were classified as “Up” when increase was >30%, “Same” if change was smaller than ±30%, or “Down” if decrease was >30%.

**Figure 3.**
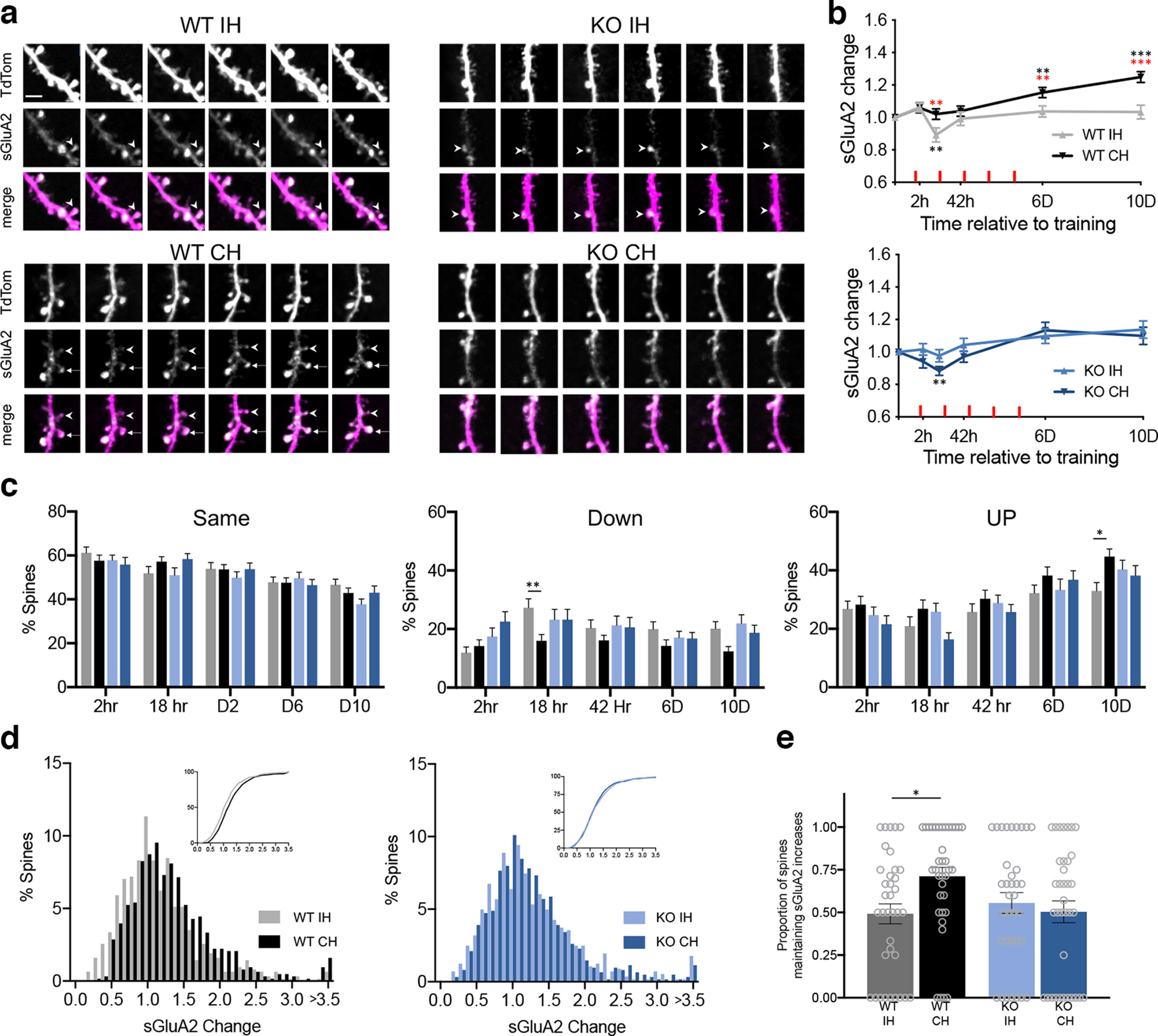
Motor skill training results in accumulation of sGluA2 in the contralateral hemisphere of WT but not *Fmr1* KO mice. ***a***, Images of sGluA2 changes. Arrows and arrowheads point to spines with persistent and transient increases in sGluA2, respectively. Scale bar = 5 um. ***b***, sGluA2 changes in persistent spines. WT-IH (gray, 614 spines, 36 dendrites), WT-CH (black, 630 spines, 37 dendrites), KO-IH (light blue, 616 spines, 35 dendrites). KO-CH (dark blue, 564 spines, 36 dendrites). *n* = 5 mice per group. Geometric mean ± SEM. Nested random effects ANOVA model. Genotype × Hemisphere × Time: *F*_(4,11941)_ = 5.38, *p* = 0.003. Red stars indicate CH versus IH comparisons and black stars indicate T0hr versus T_i_. *p < 0.05, **p < 0.01, and ***p < 0.001. ***c***, Proportion of persistent WT and KO spines with increased (Up), decreased (Down) and unchanged (Same) levels of sGluA2. WT-IH *n* = 36 dendrites, WT-CH *n* = 37 dendrites, KO-CH *n* = 36 dendrites and KO-IH *n* = 35 dendrites. Three-way ANOVA with *post hoc* one-way ANOVA and Sidak correction, Same: Time, *F*_(4,700)_ = 20.71, *p* < 0.0001; Down: Genotype × Hemisphere, *F*_(1,699)_ = 5.45, *p* = 0.02; Up: Time, *F*_(4,700_ = 22.55, *p* < 0.0001; Genotype × Hemisphere, *F*_(1,700)_ = 11.42, *p* < 0.001. *p < 0.05 and **p < 0.01. ***d***, Distribution histograms and cumulative distributions (insets) of average D6 and D10 sGluA2 change. WT: *p* < 0.0001, KO *p* = 0.44 (Kolmogorov–Smirnov test). ***e***, Proportion of spines with increased sGluA2 at 18 h that maintain the increase until day 10. N = dendrite. Two-way ANOVA with Sidak correction, Genotype, *F*_(1,139)_ = 5.28, *p* = 0.2; Hemisphere, *F*_(1,139)_ = 2.03, *p* = 0.16; Genotype × Hemisphere, *F*_(1,139)_ = 5.28 *p* = 0.02, *p < 0.05 See Extended Data Figures 3-1, 3-2, 3-3, 3-4, and 3-5 and Extended Data Table 3-1 for additional information.

10.1523/ENEURO.0364-22.2023.f3-1 Extended Data Figure 3-1 Basal levels of sGluA2 in spines is lower in the *Fmr1* KO mice. Cumulative distributions of sGluA2 at T0 in contralateral and ipsilateral hemispheres of WT and *Fmr1* KO mice. Download Figure 3-1, TIF file.

10.1523/ENEURO.0364-22.2023.f3-2 Extended Data Figure 3-2 Normalized geometric mean of dendritic shaft tdTomato intensity in IH and CH hemispheres of WT and KO mice. Download Figure 3-2, TIF file.

10.1523/ENEURO.0364-22.2023.f3-3 Extended Data Figure 3-3 Raster plots of percent sGluA2 change relative to T0 at different times following training in IH and CH of WT and KO mice. Spines are sorted by D10 changes. Download Figure 3-3, TIF file.

**Figure 4.**
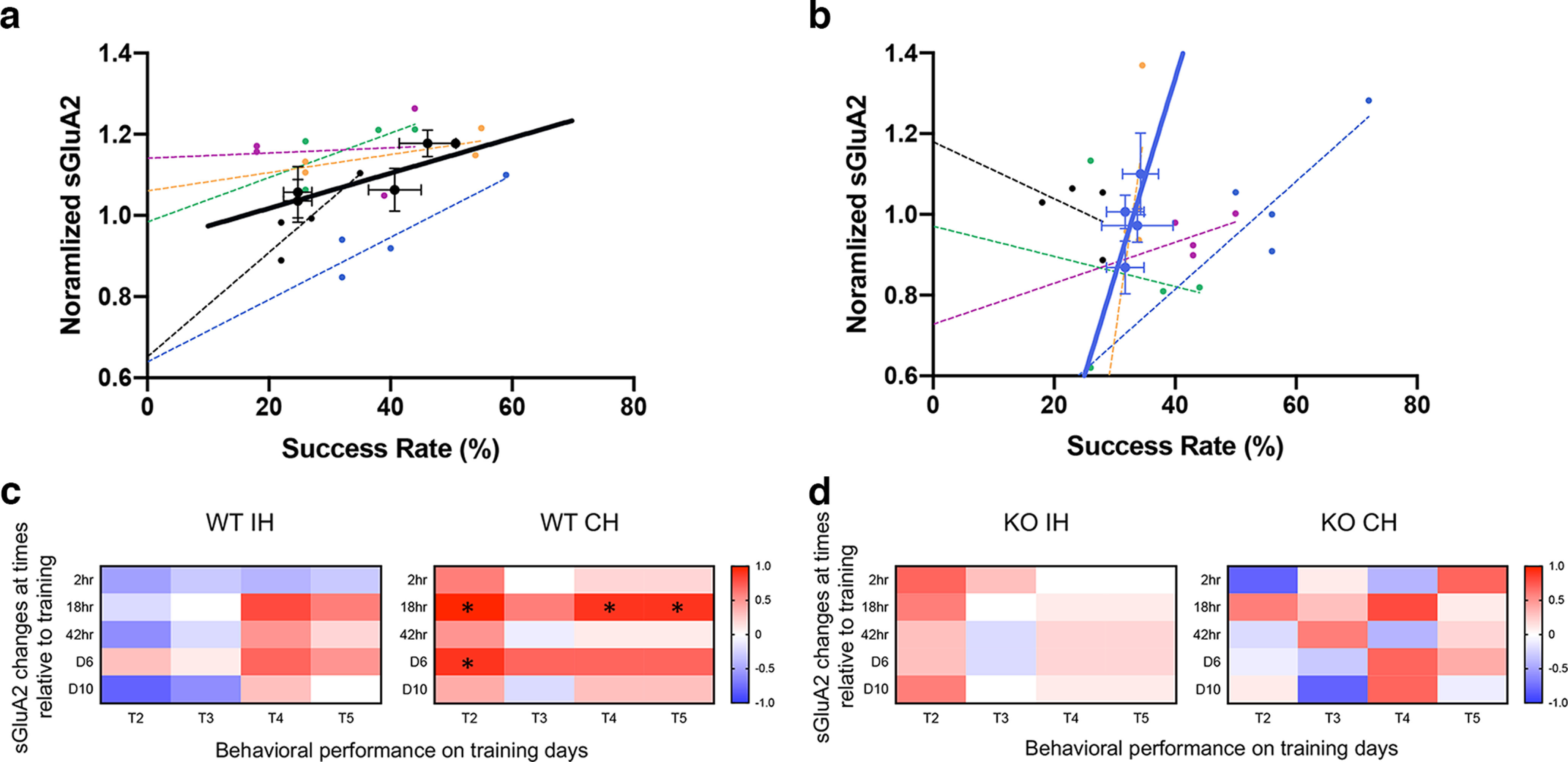
sGluA2 changes in contralateral hemisphere of WT mice correlate with Increased behavioral performance. ***a***, Correlation of reaching performance and sGluA2 levels at individual training sessions for which imaging was performed 2–24 h thereafter. Small symbols represent individual mice, and dashed lines are linear regressions for each mouse. Bold blue symbols represent average sGluA2 levels and behavioral performance of *n* = 5 mice at each imaging session for WT and Fmr1 KO (***b***) mice. Bold line is the linear regression for the average values (WT, *R*^2^ = 0.75, *p* = 0.13; KO, *R*^2^ = 0.46, *p* = 0.32). Error bars, SEM. Correlations (Spearman coefficient) between sGluA2 changes at different time points and behavioral performance on different days in (***c***) WT and (***d***) KO mice. *p < 0.05. See Extended Data Figure 4-1 for additional information.

10.1523/ENEURO.0364-22.2023.f3-4 Extended Data Figure 3-4 Spines intensity changes WT-IH (grey, 614 spines, 36 dendrites), WT-CH (black, 631 spines, 37 dendrites), KO-IH (light blue, 616 spines, 35 dendrites). KO-CH (dark blue, 564 spines, 36 dendrites). *n* = 5 mice per group. Geometric mean ± SEM. Nested random effects ANOVA model. Genotype × Hemisphere × Time, *F*_(4,12055)_ = 1.54, *p* = 0.5085. Red stars indicate CH versus IH comparisons and black stars indicate T0h versus T_i_. *p < 0.05, **p < 0.01, and ***p < 0.001. ***b***, Correlation between average (D6 and D10) spine size and spine sGluA2 change with linear fit. Pearson *R* values are indicated. Download Figure 3-4, TIF file.

10.1523/ENEURO.0364-22.2023.f3-5 Extended Data Figure 3-5 Proportion of spines with increased sGluA2 at 18 h that maintain the increase until day 10 in untrained WT mice (WT). N = dendrite. Two-way ANOVA with Sidak correction, Genotype, *F*_(1,139)_ = 5.28, *p* = 0.2; Hemisphere, *F*_(1,139)_ = 2.03, *p* = 0.16; Genotype × Hemisphere, *F*_(1,139)_ = 5.28 *p* = 0.02, *p < 0.05. Download Figure 3-5, TIF file.

10.1523/ENEURO.0364-22.2023.f3-6 Extended Data Figure 3-6 sGluA2 levels in the WT-IH (grey), WT-CH (black), KO-IH (light blue), and KO-CH (dark blue) hemispheres in four groups based on increasing percentile rank of sGluA2 intensity in spines at T0. Number of spines in each group (IH/CH) are indicated in the legend. Geometric means ± SEM. Nested random effects ANOVA model. Genotype × Hemisphere × Time, *F*_(4,11948)_ = 5.67, *p* = 0.0001; Genotype × Hemisphere × Group, *F*_(3,11948)_ = 5.81, *p* = 0.0006; Hemisphere × Time, *F*_(4,11948)_ = 8.58, *p* < 0.0001; Hemisphere × Group, *F*_(3,11948)_ = 5, *p* = 0.002. Stars indicate T0 h versus Ti for Group 1. **p < 0.01. Download Figure 3-6, TIF file.

10.1523/ENEURO.0364-22.2023.tab3-1 Extended Data Table 3-1 Full statistical information for Figure 3. Download Table 3-1, DOCX file.

##### Correlation of sGluA2 change and spine formation with reaching performance

For Figure 4*a*,*b* and Extended Data Figure 4-1*a*,*b*, imaging data from the CH at 2-h, 18-h, 42-h, and 6-d time points was correlated with the performance on the closest previous training day (day1 for 2- and 18-h, day 2 for 42-h, and day 5 for the 6-d imaging time). Linear regression lines were computed for each mouse as well as for the average of WT or KO mice as in (Roth et al., 2020). For Figure 4*c*,*d* and Extended Data Figure 4-1*c*,*d*, Spearman correlations for sGluA2 change or spine formation for every imaging session and behavioral performance on all five training days was calculated using MATLAB’s correlation (corr) function.

10.1523/ENEURO.0364-22.2023.f4-1 Extended Data Figure 4-1 Spine formation in contralateral hemisphere of WT mice does not correlate with increased behavioral performance. ***a***, Correlation of reaching performance and spine formation at individual training sessions for which imaging was performed 2–24 h thereafter. Small symbols represent individual mice, and dashed lines are linear regressions for each mouse. Bold blue symbols represent average spine formation and behavioral performance of *n* = 5 mice at each imaging session for WT and *Fmr1* KO (***b***) mice. Bold line is the linear regression for the average values WT (*R*^2^ = 0.07, *p* = 0.74; KO, *R*^2^ = 0.01, *p* = 0.88). Error bars, SEM. Correlations (Spearman coefficient) between spine formation at different time points and behavioral performance on different days in (***c***) WT and (***d***) KO mice. Download Figure 4-1, TIF file.

##### Statistics

Analysis was done either on GraphPad prism or Proc GLIMMIX from SAS/STAT Software and error bars represent standard error means (SEM). To test for statistical significance Student’s *t* test, Kolmogorov–Smirnov test, one-way ANOVA, two-way ANOVA, two-way repeated measures ANOVA or three-way ANOVA with Bonferroni or Sidak multiple comparison correction was used. A nested random effects ANOVA model (observations nested within dendrites and further nested within mice) with unequal variance components with corrections calculated by simulation techniques were also used. To improve readability and because of the large number of statistical comparisons across time, hemispheres and genotypes, many statistics values were not included in the main text but mentioned in the legends and all are included in Extended Data Tables 1-1, 2-1, and 3-1.

## Results

### Impaired motor skill learning in *Fmr1* KO mice following utero electroporation

To track spine and AMPAR dynamics in L2/3 pyramidal neurons of M1 cortex following motor skill training, E15.5 mouse embryos were electroporated *in utero* with AMPAR subunit GluA2 tagged to superecliptic phluorin (sGluA2), a pH sensitive form of GFP, and tdTomato as a morphologic tracer. Cranial windows were implanted, and images were collected starting from P49 before, during, and after training on a forelimb-reaching task in anesthetized mice. As previously described (Suresh and Dunaevsky, 2017), transfected neurons had high expression of sGluA2 in spines throughout the dendritic arbor, with relatively lower expression in dendritic shafts (Fig. 1*b*). Littermate control (WT) mice improved over the 5-d training period, almost doubling their success rate on the fifth training session (Fig. 1*c*; Extended Data Fig. 1-1*a*; Extended Data Table 1-1). In contrast to WT mice, the *Fmr1* KO mice did not display much improvement with training although the number of reaches performed by the KO mice was similar or higher to WT mice (Extended Data Fig. 1-1*b*; Extended Data Table 1-1). Thus, we concluded that motor skill learning occurs in WT mice following *in utero* electroporation and confirmed the motor-skill learning deficit in the *Fmr1* KO mouse.

### Transient motor skill training-induced dendritic spine formation is observed in both WT and the *Fmr1* KO mouse

Previously, it was shown that training-induced spine formation in L5 neurons was absent in the *Fmr1* KO mice (Padmashri et al., 2013). To determine whether similar deficits in training-induced spine formation are observed in L2/3 excitatory neurons in *Fmr1* KO mice, we imaged the same apical dendrites in L1 of the motor cortex before, during, and after the 5-d training period (Fig. 2*a*). We imaged both, the contralateral hemisphere (CH) and the ipsilateral hemispheres (IH) to the trained forelimb. As previously reported (Suresh and Dunaevsky, 2017), before training started we observed an increase in the density of spines in the *Fmr1* KO mice compared with the WT mice (Extended Data Fig. 2-1). In the WT mice CH, similar to L5 pyramidal neurons, motor skill training resulted in a rapid but transient increase in total number of spines that peaked (∼15%) at 18 h after training (Fig. 2*b*). Surprisingly, an increase in total number of spines (∼8%), albeit trending smaller than WT, was also observed in the *Fmr1* KO mouse (Fig. 2*b*). Overall, no significant differences in total spines were observed between the genotypes (Extended Data Table 2-1). The transient increase in spine density in the CH in the WT mouse was because of a significant increase in spine formation during the first days of training, which was followed by a delayed increase in spine elimination (Fig. 2*c*). The increase in total spines in the KO CH is because of a combination of increase in formation and decrease in elimination although individually they are not significantly different from KO IH formation and elimination values (Fig. 2*c*). These data demonstrate that motor skill learning results in transient increase in spine density in both WT and KO mice.

Previously, it was demonstrated that with motor skill training, newly formed spines were more likely to persist in both WT and *Fmr1* KO mice (Xu et al., 2009; Reiner and Dunaevsky, 2015). To assess stability of new spines in L2/3 neurons, we identified new spines at 2 and 18 h following first day of training and determined the proportion of new spines that were present on D10. In L2/3 spines, we only observed a trend toward increased stability of new spines in the CH in both the WT and *Fmr1* KO mice (Fig. 2*d*). Combined these data suggest that formation and stabilization of new spines in L2/3 neurons with motor skill training are similar in in the WT and KO mice and therefore unlikely to explain the behavioral deficit in the *Fmr1* KO mice.

### Impaired accumulation of AMPAR with motor skill training in the *Fmr1* KO mouse

Altered GluA2 dynamics in untrained *Fmr1* KO mice was previously demonstrated (Suresh and Dunaevsky, 2017). It was also shown that motor skill learning results in accumulation of the GluA1 subunit of AMPAR in dendritic spines (Roth et al., 2020), but whether there is an impairment in experience-dependent plasticity of AMPAR in FXS is not known. Spine-surface GluA2 levels were measured by the mean SEP fluorescence (sGluA2) in spines. As previously described (Suresh and Dunaevsky, 2017), we found that at baseline spines in the *Fmr1* KO mouse had less sGluA2 levels as compared with the WT in both the ipsilateral and contralateral hemispheres (Extended Data Fig. 3-1). We next determined how motor skill training modified the surface AMPARs in dendritic spines of *Fmr1* KO and littermate control mice (Fig. 3*a*). We quantified spine-surface GluA2 levels by normalizing the mean SEP fluorescence (sGluA2) in spines to the mean tdTomato fluorescence on the adjacent dendritic shaft. Dendritic tdTomato fluorescence was relatively constant over the experimental time-period in WT mice, making it an acceptable normalization factor for small variations in daily imaging conditions (Extended Data Fig. 3-2; Cane et al., 2014; Suresh and Dunaevsky, 2017; Roth et al., 2020). sGluA2 changes were expressed relative to the first day of imaging and ranged from reductions of up to 0.007 to 9-fold increases across all four groups (Extended Data Fig. 3-3). In the CH of WT mice, we observed an increase of ∼20% in sGluA2 in persistent spines that was significant at D6 and D10 (Fig. 3*a*,*b*; Extended Data Table 3-1). Interestingly, unlike the transient change in spine numbers, the sGluA2 change not only persisted after 5 d of motor skill training has ended, but also continued to increase. These changes were dependent on motor skill training as similar to untrained mice (Suresh and Dunaevsky, 2017) no significant increase in sGluA2 was observed in the IH of trained mice (Fig. 3*b*). In contrast, motor skill training did not significantly increase sGluA2 in either hemisphere of the *Fmr1* KO mouse (Fig. 3*a*,*b*; Extended Data Table 3-1). Transient, small but significant decreases in sGluA2 were also observed at 18 h following first day of training in IH WT and CH KO. In the CH, sGluA2 changes were significantly different between the WT and *Fmr1* KO mice at 18 h and D10 (Extended Data Table 3-1). We also measured spine size changes by quantifying the tdTomato fluorescence in spines. Although spine intensity changes were highly correlated with sGluA2 changes (Extended Data Fig. 3-4), we observed no corresponding increase in mean spine size in the CH of WT mice. Rather, small but significant decreases were observed in all other conditions (Extended Data Fig. 3-4) similar to what was reported in untrained mice (Suresh and Dunaevsky, 2017). These data indicate that motor skill training results in sustained increase of sGluA2 in WT mice that is impaired in the *Fmr1* KO mice.

It was previously shown, in untrained mice, that GluA2 levels within spines are highly dynamic; with spines showing increases, decreases or no change between days of imaging (Suresh and Dunaevsky, 2017). The same was observed for both IH and CH of both WT and KO mice, with spines showing diverse changes in GluA2 levels from day to day (Fig. 3*c*). Since the proportion of spines with no sGluA2 changes (Same, defined as <30% change) was not different between the hemispheres, the total increase in sGluA2 in the WT CH was driven by a combination of decreased proportion of spines showing loss of sGluA2 (Down) and increased proportion of spines accumulating sGluA2 (Up). During early stages of training (18 h) the interhemispheric change in sGluA2 levels is mainly driven by fewer (40% decrease) spines showing a reduction in sGluA2 levels in the CH (Down in Fig. 3*c*). However, the proportion of spines with accumulated sGluA2 was 40% higher in the CH by D10. Moreover, the distribution of sGluA2 changes in spines on the last 2 d (average of D6 and D10) was significantly shifted toward larger increases in the CH of WT but not KO mice (Fig. 3*d*). Interestingly, in the CH of WT mice, the spines that had increases in sGluA2 at 18 h following training were also 40% more likely to maintain this increase even 5 d after the end of training (D10). This was not observed in the IH of trained mice, in untrained WT mice (Extended Data Fig. 3-5), or in either hemisphere of trained *Fmr1* KO mice (Fig. 3*e*). Thus, both reduced loss and increased gain in sGluA2 contribute to the increased sGluA2 levels with training in the WT mice and early gains in sGluA2 in dendritic spines are selectively stabilized in the CH.

To better understand which population of persistent spines contributes to the total increase in sGluA2 with training in WT mice, we categorized spines into four groups based on their initial relative sGluA2 levels (see Materials and Methods). Since smaller spines are less stable, the number of persistent spines in each group was not equal (Extended Data Fig. 3-6). We found that in WT CH spines with the least sGluA2 (group 1) had significant increases already detected 2 h following training with further increases at D6 and D10 (Extended Data Fig. 3-6; Extended Data Table 3-1). No significant changes in sGluA2 were observed soon after training begun in the rest of the spines, although spines with intermediate sGluA2 levels (groups 2 and 3) in the CH did display significant relative increases by the last day of imaging (Extended Data Fig. 3-6). In the KO mice, smaller increases in the smallest persistent spines (group 1) were observed in both hemispheres but these were not significant early on (Extended Data Fig. 3-6; Extended Data Table 3-1). Although the smallest persistent spines (group 1) were previously shown to have increases (∼50%) in sGluA2 over time in both WT and KO untrained mice (Suresh and Dunaevsky, 2017), with training, the increases in the WT CH were 2-fold larger and started earlier. Thus, GluA2 accumulation with training in WT mice occurs in both small and large spines with the smallest spines having the largest relative increases.

Finally, we examined the relationship between sGluA2 changes and motor skill task performance. We first correlated the performance to the sGluA2 changes observed within 2–24 h after training days (Fig. 4*a*,*b*). In the WT mice, sGluA2 changes in the CH were all positively correlated with performance while that was not the case in all KO mice. Daily averages show that in WT trained mice performance and spine sGluA2 levels increased in a moderately correlated manner (*R*^2^ = 0.75, *p* = 0.13). This relationship was much less correlated in the KO mice (*R*^2^ = 0.46, *p* = 0.32). None of the correlations reached significance, likely because of the use of only four time points for this analysis, as we did not image after each training session. We next correlated the average sGluA2 change at each imaging day to the success rate at each of training days and computed the Spearman correlation (Fig. 4*c*,*d*). In the WT mice, sGluA2 changes in the CH were positively correlated with performance at most training days. Significant correlations were observed between sGluA2 changes at 18 h after the first day of training and performance on training days 2, 4, and 5 as well as sGluA2 changes at 42 h after training and performance on day 6. No clear pattern of correlations was observed in the WT IH or in either of the hemispheres in the KO mice, and none were statistically significant. We performed a similar analysis correlating spine formation in the CH with performance and found no correlation in WT mice (*R*^2^ = 0.07, *p* = 0.74) or KO mice (*R*^2^ = 0.01, *p* = 0.88; Extended Data Fig. 4-1*a*,*b*). Similarly, no significant correlations were observed between average spine formation in the CH at each imaging day and the success rate at each of training days in either WT or KO mice (Extended Data Fig. 4-1*c*,*d*). Overall, our findings indicate that motor skill training drives increases in surface AMPAR subunit expression in persistent dendritic spines and that performance is best correlated with early sGluA2 increases in the CH. However, this synaptic strengthening is impaired in the *fmr1* KO mouse.

## Discussion

Here, we set out to determine how motor skill training alters AMPAR within dendritic spines in the primary motor cortex and whether there are deficits in the fragile X syndrome mouse model. We imaged a tagged GluA2 subunit of AMPA receptors in dendritic spines of L2/3 neurons following a forelimb-reaching task in WT and *Fmr1* KO mice. Our study demonstrates that unlike in L5 neurons (Padmashri et al., 2013) there is no deficit in training-induced spine formation in the L2/3 neurons of the *Fmr1* KO mice. We also report that in addition to formation of new spines, training results in accumulation of GluA2 in preexisting persistent spines, however this increase only occurs in the CH of WT mice and not in the *Fmr1* KO mice. We conclude that in L2/3 neurons accumulation of AMPAR in spines is better correlated with behavioral improvement than spine formation.

The motor skill deficit observed in the *Fmr1* KO is more severe than what was previously reported in younger (five weeks) mice (Padmashri et al., 2013). Similar severe deficit in motor skill learning was previously reported in adult *Fmr1* KO mice (Hodges et al., 2017). It is possible that increased age at which mice were trained, because of the recovery after chronic window transplantation, could be a contributing factor. While we cannot rule out that *in utero* electroporation or stress from food restriction and anesthesia could more adversely affect the *Fmr1* KO mice, similar number of attempts throughout training suggest similar level of motivation in WT and *Fmr1*KO mice. In addition, while we did not conduct an in-depth analysis of the fine movement parameters, the similar initial success rate argues against major differences in visual-motor coordination. Finally, while not examined here, it was previously shown that extending the training does not improve learning in the *Fmr1* KO mouse (Hodges et al., 2017).

We have found several laminar differences in motor skill training induced plasticity of dendritic spines. While we found similar transient spine increases with learning in L2/3 neurons of WT mice as previously reported in L5 neurons (Xu et al., 2009; Padmashri et al., 2013), we have observed reduced stabilization of the newly formed spines in L2/3 neurons (Xu et al., 2009; Reiner and Dunaevsky, 2015). The reduced stabilization could be because of the previously reported heightened spine dynamics in L2/3 compared with L5 (Chen et al., 2015; Ma et al., 2016). Surprisingly, we have found that despite a severe learning deficit in the *Fmr1* KO, we still observed a transient increase in spine numbers following motor skill training, which was not observed in L5 neurons of the *Fmr1* KO mouse (Pan et al., 2010; Padmashri et al., 2013; Reiner and Dunaevsky, 2015; Hodges et al., 2017). Thus, a correlation between training induced spine increases and learning does not hold in L2/3, as spine increases are observed in both genotypes while learning is severely impaired in the KO mice at this age. These data underscore the importance of investigating other synaptic changes besides formation of new spines. In addition, future studies should examine whether spine contact with presynaptic terminals (Harris, 2020) and astrocyte perisynaptic processes (Genoud et al., 2006) is differentially modulated with motor skill learning.

Much emphasis has been placed on the formation and stabilization of spines with motor skill learning (Xu et al., 2009; Yu and Zuo, 2011; Fu et al., 2012; Padmashri et al., 2013; Reiner and Dunaevsky, 2015). However, the spine formation data were at odds with experiments that assessed synaptic strength using electrophysiological measurements, as only 5–10% increases in spine number were observed and these increases were transient, returning to baseline at a time when synaptic strengthening in the CH was observed (Rioult-Pedotti et al., 2000, 2007; Harms et al., 2008; Padmashri et al., 2013; Padmashri and Dunaevsky, 2019). Our data demonstrate accumulation of AMPARs in persistent spines in the CH of WT mice similarly to what was reported for GluA1 in L5 neurons (Roth et al., 2020). We detected changes in GluA2 immediately after training, at a time when new spines are also formed and further increases with additional training and even after training has been completed (5 d after completion of training). In the *Fmr1* KO mice, which have a motor skill-learning deficit, training-induced GluA2 accumulation was not observed in either early or later time points. This was surprising given the observation of structural plasticity in the CH of the *Fmr1* KO and suggests a decoupling of structural and functional plasticity of synapses of L2/3 neurons and impairment in experience-dependent functional plasticity. The impaired experience dependent plasticity of AMPARs is consistent with other studies indicating deficits in long-term potentiation in the *Fmr1* KO mice (Lauterborn et al., 2007; Suvrathan et al., 2010; Yun and Trommer, 2011; Padmashri et al., 2013). Furthermore, since spine number increases mainly precede GluA2 increases, this indicates a model whereby synaptic strengthening is impaired, potentially contributing to the learning deficits in the *Fmr1* KO mice. However, the possibility of differential effect of overexpression of GluA2 on synaptic properties in the two genotypes has not been explored in our study.

In the WT mice, about half of the spines in both hemispheres did not change their levels of GluA2, and the total increase in AMPARs was because of both fewer spines losing GluA2 and more spines accumulating GluA2 in the CH. In fact we observed significant dip in GluA2 levels at 18 h after training in WT IH that was not observed in the CH nor was it previously observed in WT untrained mice (Suresh and Dunaevsky, 2017) suggesting there might be an early LTD like mechanism involved in the IH. Although, ability to induce LTD is enhanced in the CH in rats (Rioult-Pedotti et al., 2000, 2007), whether it is also partially occluded in the IH in very early stages of training is not known. Surprisingly, we observed a similar dip in sGluA2 at the same time point in the KO CH, suggesting dysregulation of synaptic plasticity in FXS motor cortex. We have also observed a decrease in spine size (as measured by tdTomato intensity) in spines of WT and *Fmr1* KO mice similar to what has been reported for untrained mice (Suresh and Dunaevsky, 2017). We do not believe it affects our finding of sGluA2 accumulation with training in the WT mice as we use dendrite rather than spine tdTomato levels for daily normalization.

In a recent study, GluA1 changes were observed in both hemispheres following forelimb motor skill training in WT mice (Roth et al., 2020). We only observe GluA2 changes in the hemisphere contralateral to the trained forelimb, consistent with multiple other studies (Rioult-Pedotti et al., 1998, 2007; Harms et al., 2008; Xu et al., 2009; Padmashri et al., 2013; Reiner and Dunaevsky, 2015). A possible explanation for this discrepancy is that in our training paradigm, after forelimb preference is established, mice are unable to switch preference and use the other forelimb, thus preventing training of both forelimbs.

The AMPAR changes in persisting spines reported here in the CH track the previously reported synaptic strengthening measure in slices following motor skill training (Rioult-Pedotti et al., 1998, 2007; Harms et al., 2008; Padmashri et al., 2013). The strengthening of preexisting synapses on L2/3 neurons is therefore better correlated with functional plasticity as well as behavioral performance than new spine formation. Moreover, given the long-lasting strengthening of synapses induced by motor learning (Rioult-Pedotti, 2007), this suggests that the GluA2 accumulation may also be long-lasting and a potential substrate for long-term storage of motor memory. The lack of training induced GluA2 insertion into spines provides an insight into the molecular mechanisms by which loss of FMRP expression results in impaired motor-skill learning.

## Synthesis

Reviewing Editor: Kristen Harris, The University of Texas at Austin Decisions are customarily a result of the Reviewing Editor and the peer reviewers coming together and discussing their recommendations until a consensus is reached. When revisions are invited, a fact-based synthesis statement explaining their decision and outlining what is needed to prepare a revision will be listed below. The following reviewer(s) agreed to reveal their identity: NONE.

Before this paper can be sent out for review, most of the graph figures need example raw images to support them. Figure 1 is nice, but insufficient to evaluate the outcomes of the graphs.

Once the revised manuscript is received we will send it out for review.
