# Supplementary figures and images for: Impaired AMPARs Translocation into Dendritic Spines with Motor Skill Learning in the Fragile X Mouse Model
Source: eNeuro. 2023 Mar 24;10(3):ENEURO.0364-22.2023. doi: 10.1523/ENEURO.0364-22.2023 (PMC10056836; doi:10.1523/ENEURO.0364-22.2023)

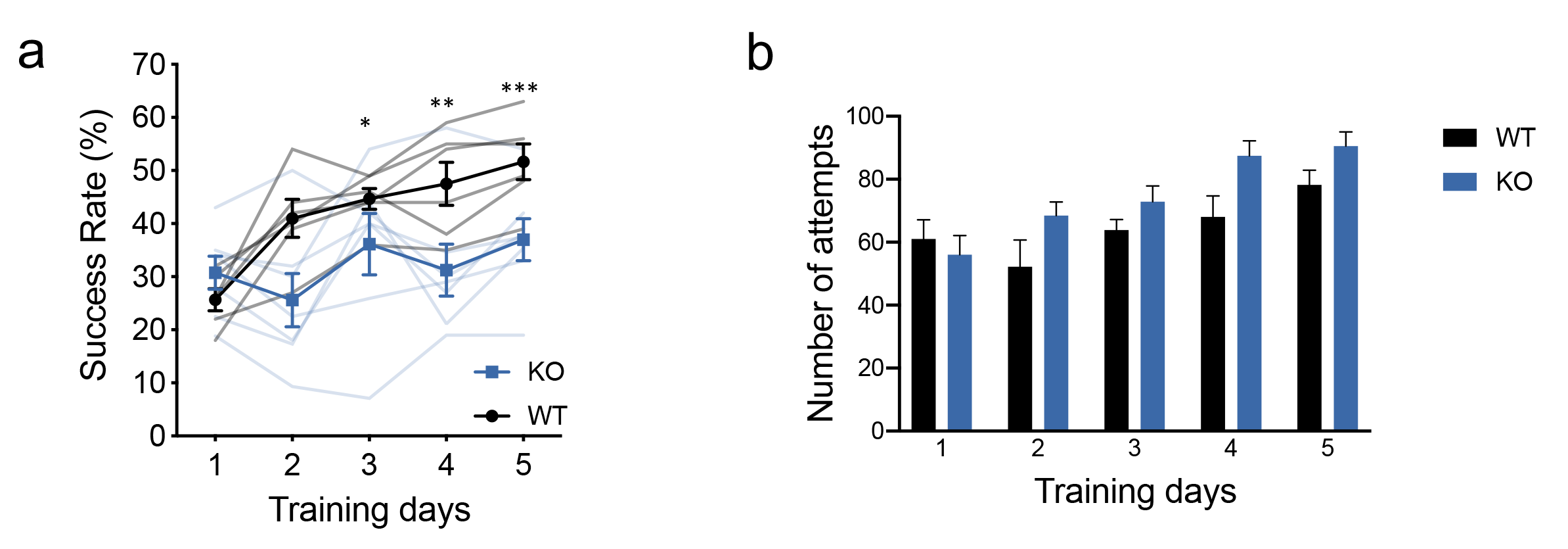

Supplement: Extended Data Figure 1-1 — Motor skill learning and number of attempts. a, Behavioral performance (success rate) of mice trained on forelimb reaching task. Thin lines represent individual mice and the bold line is the average [Fmr1 KO, blue, n = 6 mice and litter mate control (WT) black, n = 7 mice] mean ± SEM. Two-way repeated measures ANOVA followed by one-way ANOVA with Bonferroni correction. Genotype × Time, F(4,44) = 6.07, p = 0.0006. b, Number of attempts performed. Genotype × Time, F(4,44) =1.916, p = 0.12 Download Figure 1-1, TIF file. [file enu-eN-NWR-0364-22-s02.tif]

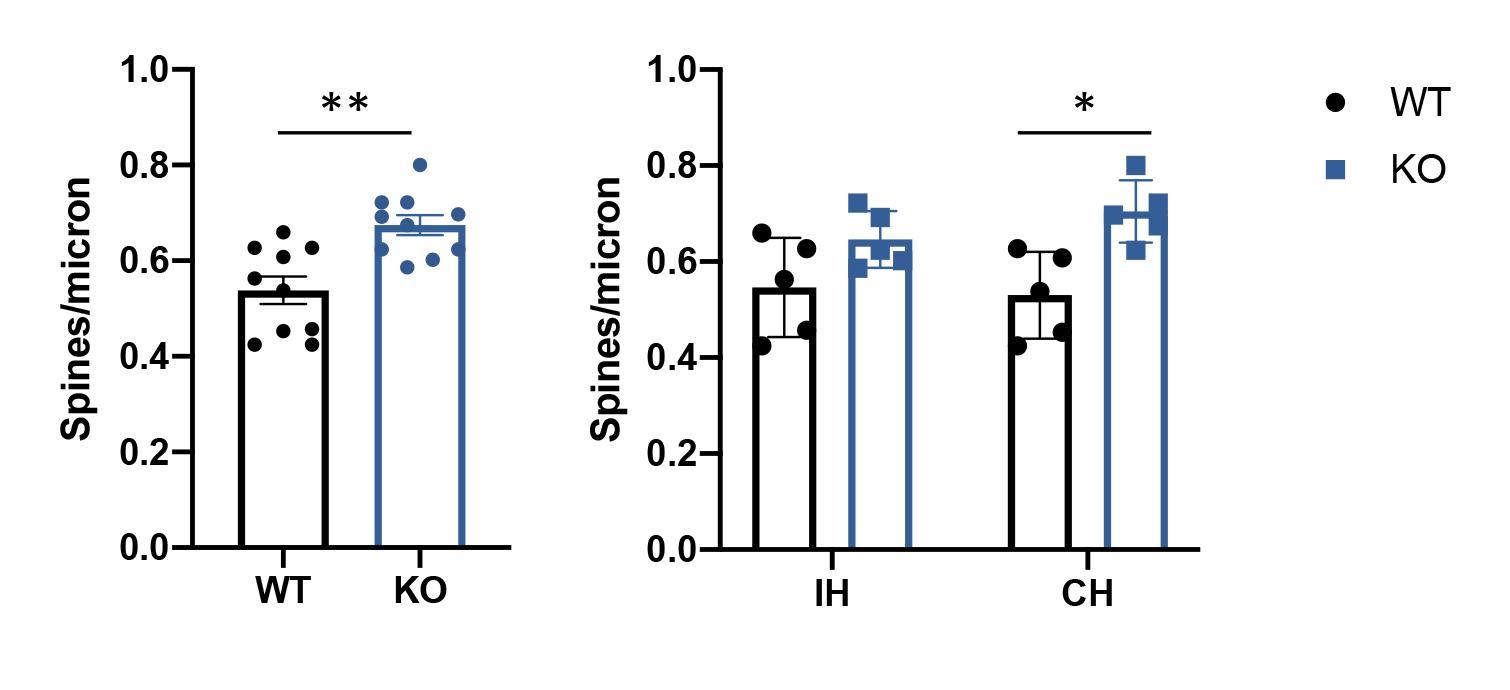

Supplement: Extended Data Figure 2-1 — Increased basal level spine density in the Fmr1 KO mouse. Download Figure 2-1, TIF file. [file enu-eN-NWR-0364-22-s03.tif]

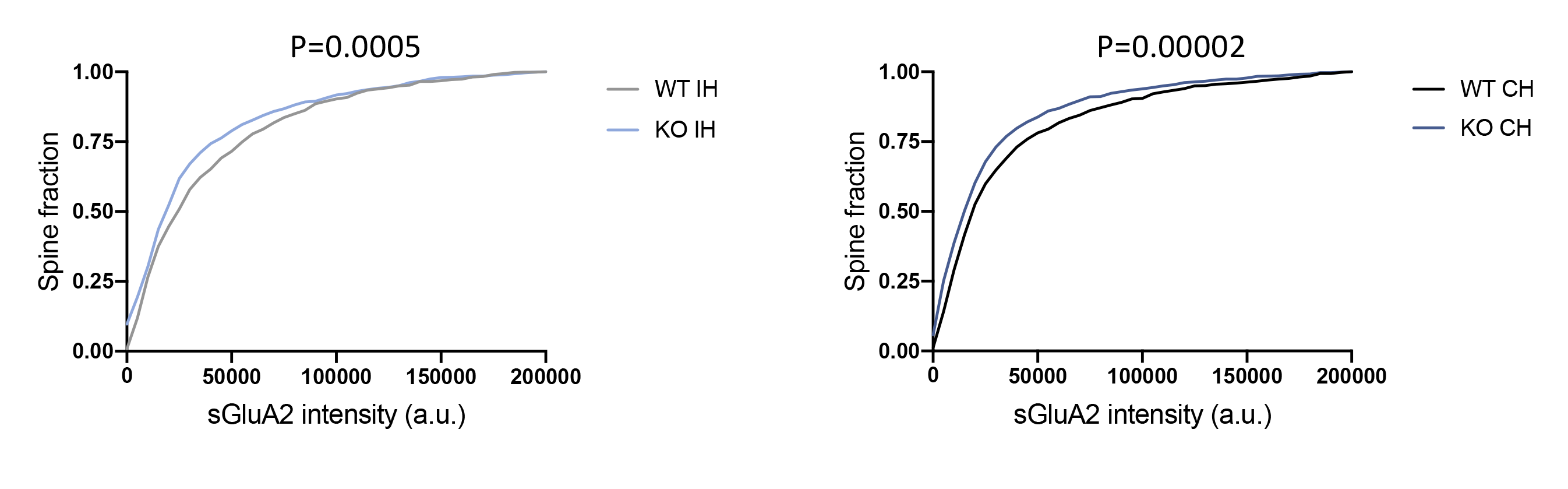

Supplement: Extended Data Figure 3-1 — Basal levels of sGluA2 in spines is lower in the Fmr1 KO mice. Cumulative distributions of sGluA2 at T0 in contralateral and ipsilateral hemispheres of WT and Fmr1 KO mice. Download Figure 3-1, TIF file. [file enu-eN-NWR-0364-22-s04.tif]

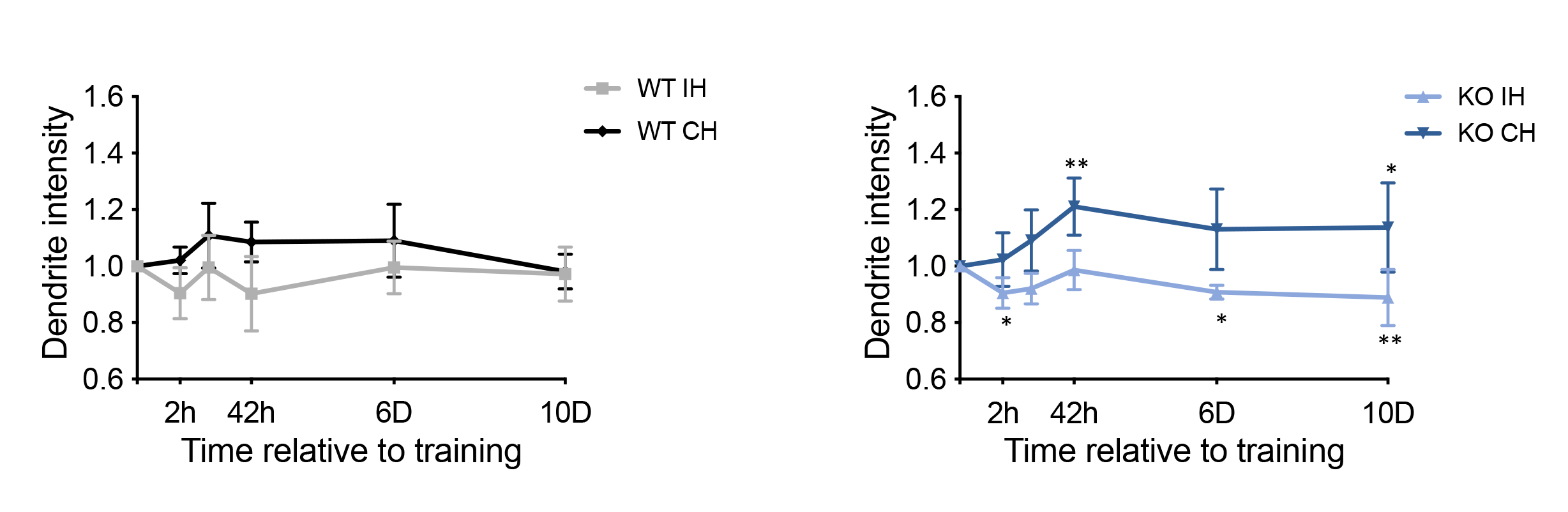

Supplement: Extended Data Figure 3-2 — Normalized geometric mean of dendritic shaft tdTomato intensity in IH and CH hemispheres of WT and KO mice. Download Figure 3-2, TIF file. [file enu-eN-NWR-0364-22-s05.tif]

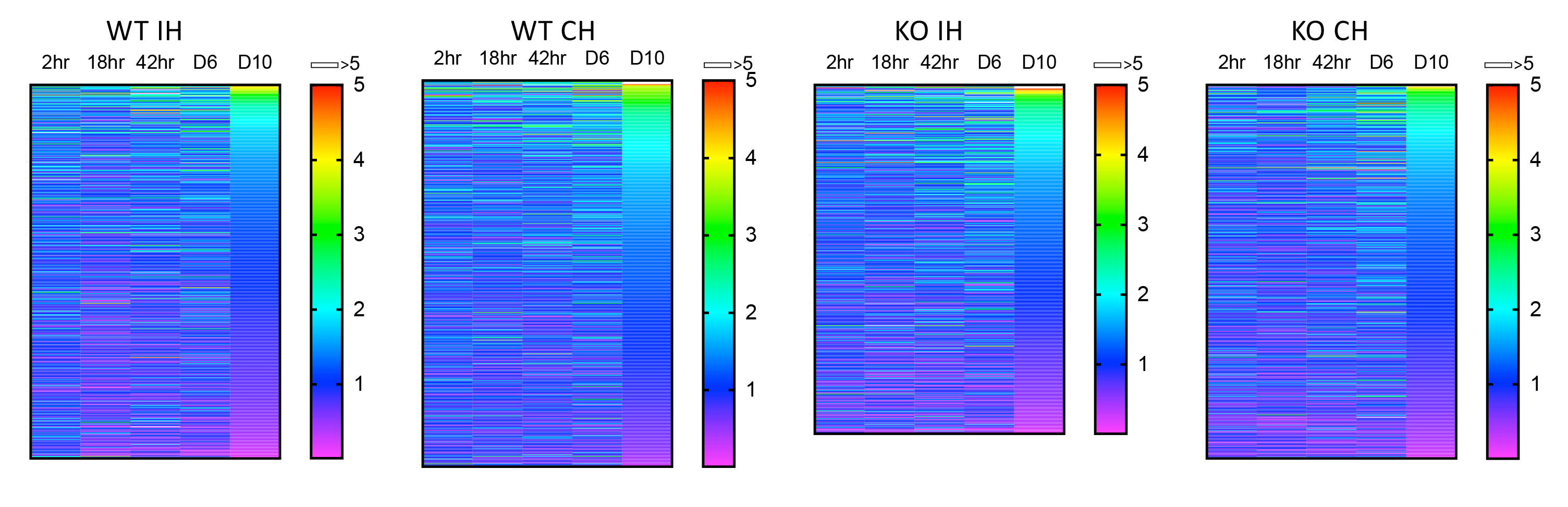

Supplement: Extended Data Figure 3-3 — Raster plots of percent sGluA2 change relative to T0 at different times following training in IH and CH of WT and KO mice. Spines are sorted by D10 changes. Download Figure 3-3, TIF file. [file enu-eN-NWR-0364-22-s06.tif]

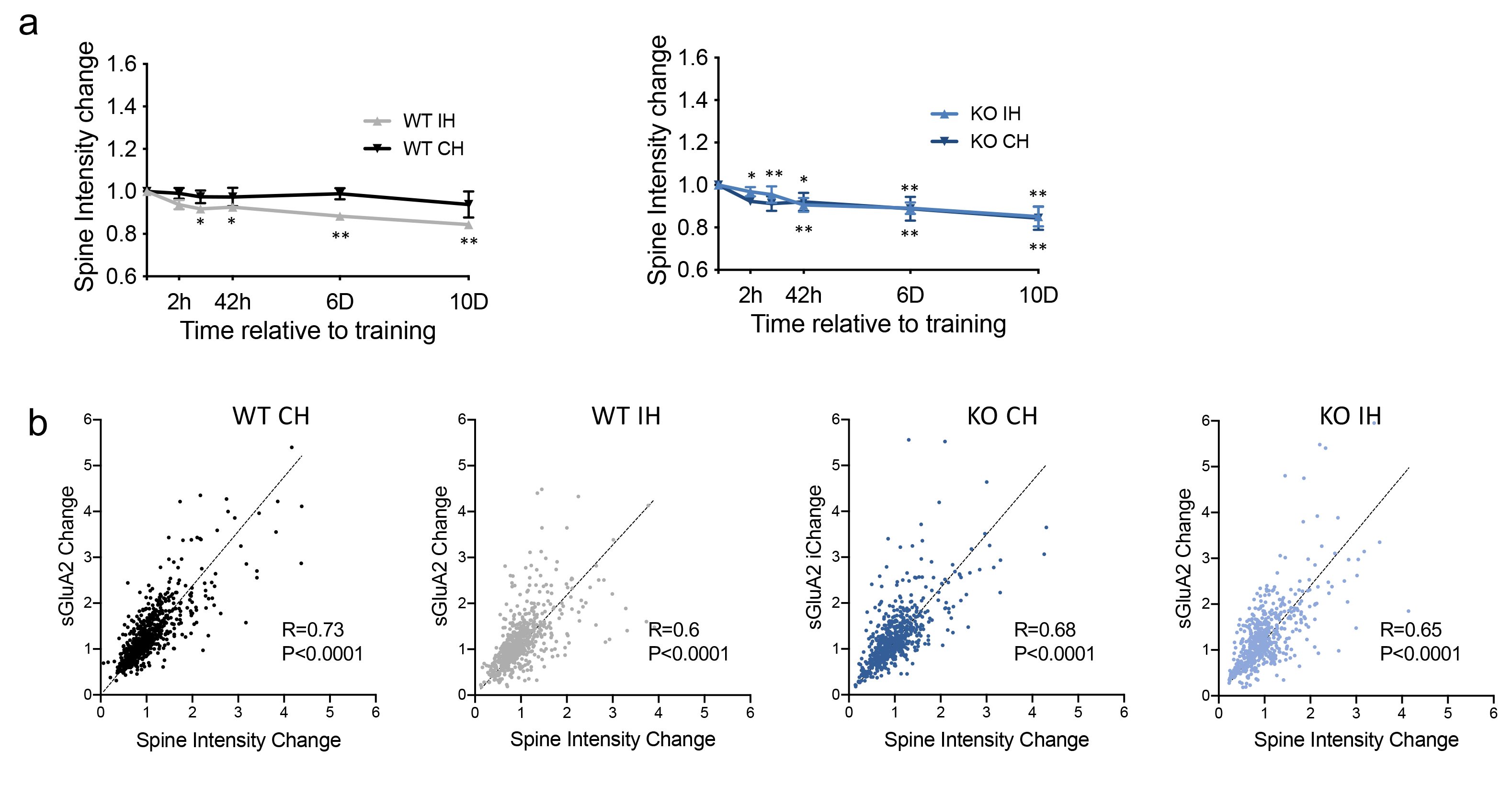

Supplement: Extended Data Figure 3-4 — Spines intensity changes WT-IH (grey, 614 spines, 36 dendrites), WT-CH (black, 631 spines, 37 dendrites), KO-IH (light blue, 616 spines, 35 dendrites). KO-CH (dark blue, 564 spines, 36 dendrites). n = 5 mice per group. Geometric mean ± SEM. Nested random effects ANOVA model. Genotype × Hemisphere × Time, F(4,12055) = 1.54, p = 0.5085. Red stars indicate CH versus IH comparisons and black stars indicate T0h versus Ti. *p < 0.05, **p < 0.01, and ***p < 0.001. b, Correlation between average (D6 and D10) spine size and spine sGluA2 change with linear fit. Pearson R values are indicated. Download Figure 3-4, TIF file. [file enu-eN-NWR-0364-22-s07.tif]

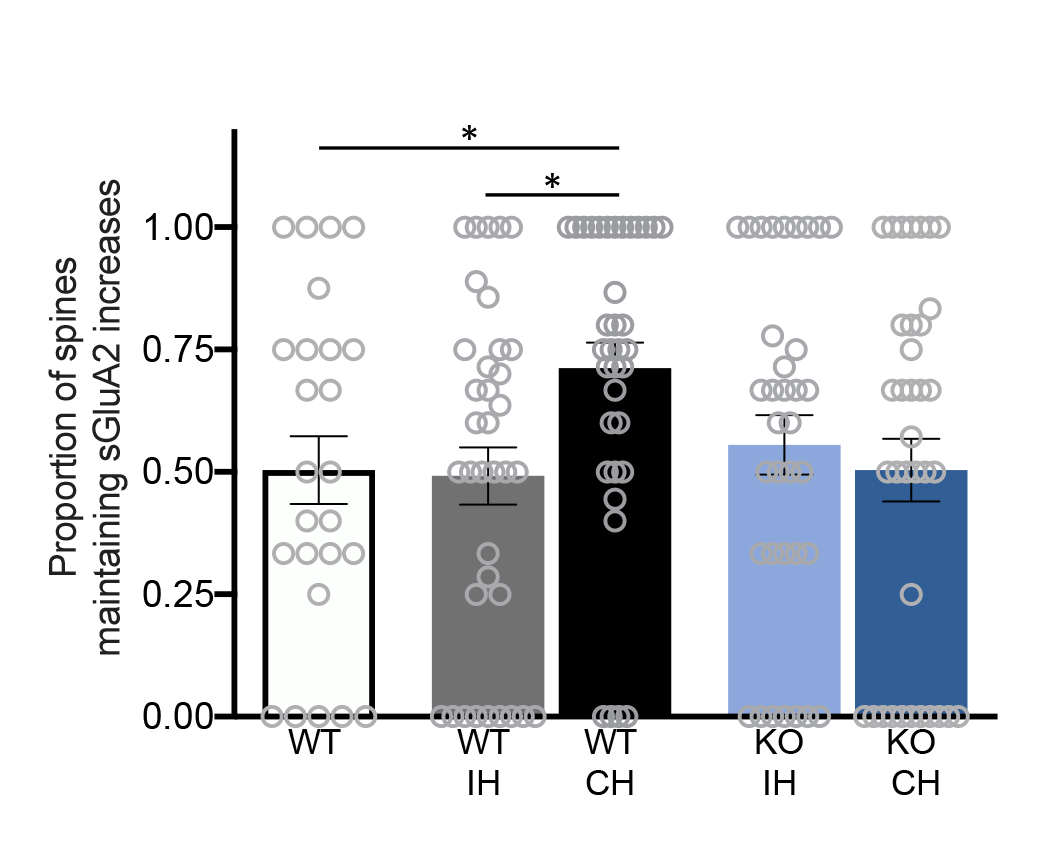

Supplement: Extended Data Figure 3-5 — Proportion of spines with increased sGluA2 at 18 h that maintain the increase until day 10 in untrained WT mice (WT). N = dendrite. Two-way ANOVA with Sidak correction, Genotype, F(1,139) = 5.28, p = 0.2; Hemisphere, F(1,139) = 2.03, p = 0.16; Genotype × Hemisphere, F(1,139) = 5.28 p = 0.02, *p < 0.05. Download Figure 3-5, TIF file. [file enu-eN-NWR-0364-22-s08.tif]

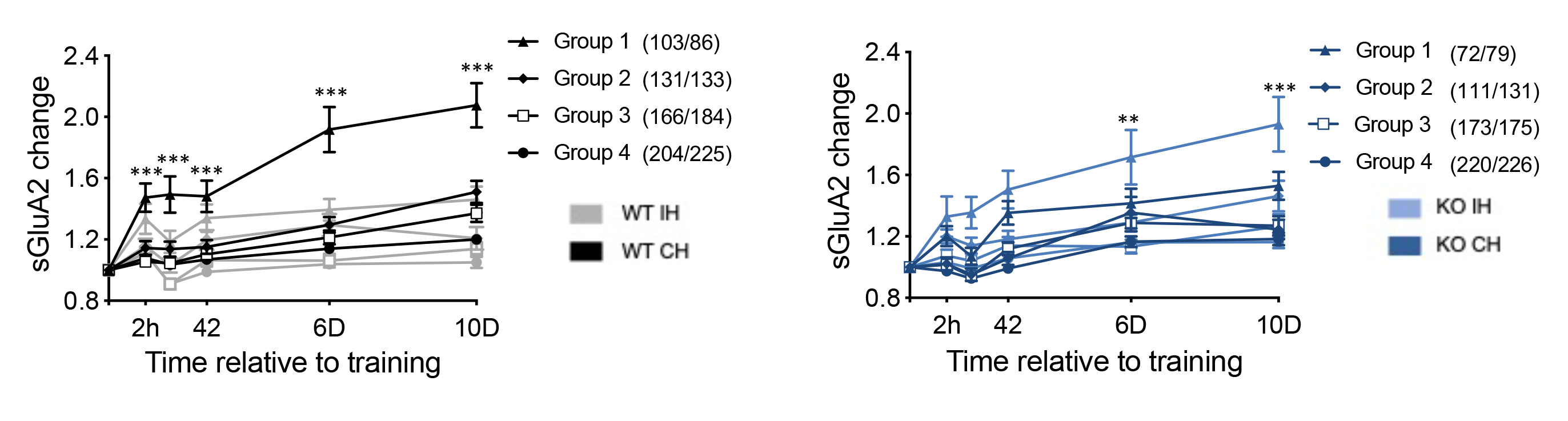

Supplement: Extended Data Figure 3-6 — sGluA2 levels in the WT-IH (grey), WT-CH (black), KO-IH (light blue), and KO-CH (dark blue) hemispheres in four groups based on increasing percentile rank of sGluA2 intensity in spines at T0. Number of spines in each group (IH/CH) are indicated in the legend. Geometric means ± SEM. Nested random effects ANOVA model. Genotype × Hemisphere × Time, F(4,11948) = 5.67, p = 0.0001; Genotype × Hemisphere × Group, F(3,11948) = 5.81, p = 0.0006; Hemisphere × Time, F(4,11948) = 8.58, p < 0.0001; Hemisphere × Group, F(3,11948) = 5, p = 0.002. Stars indicate T0 h versus Ti for Group 1. **p < 0.01. Download Figure 3-6, TIF file. [file enu-eN-NWR-0364-22-s09.tif]

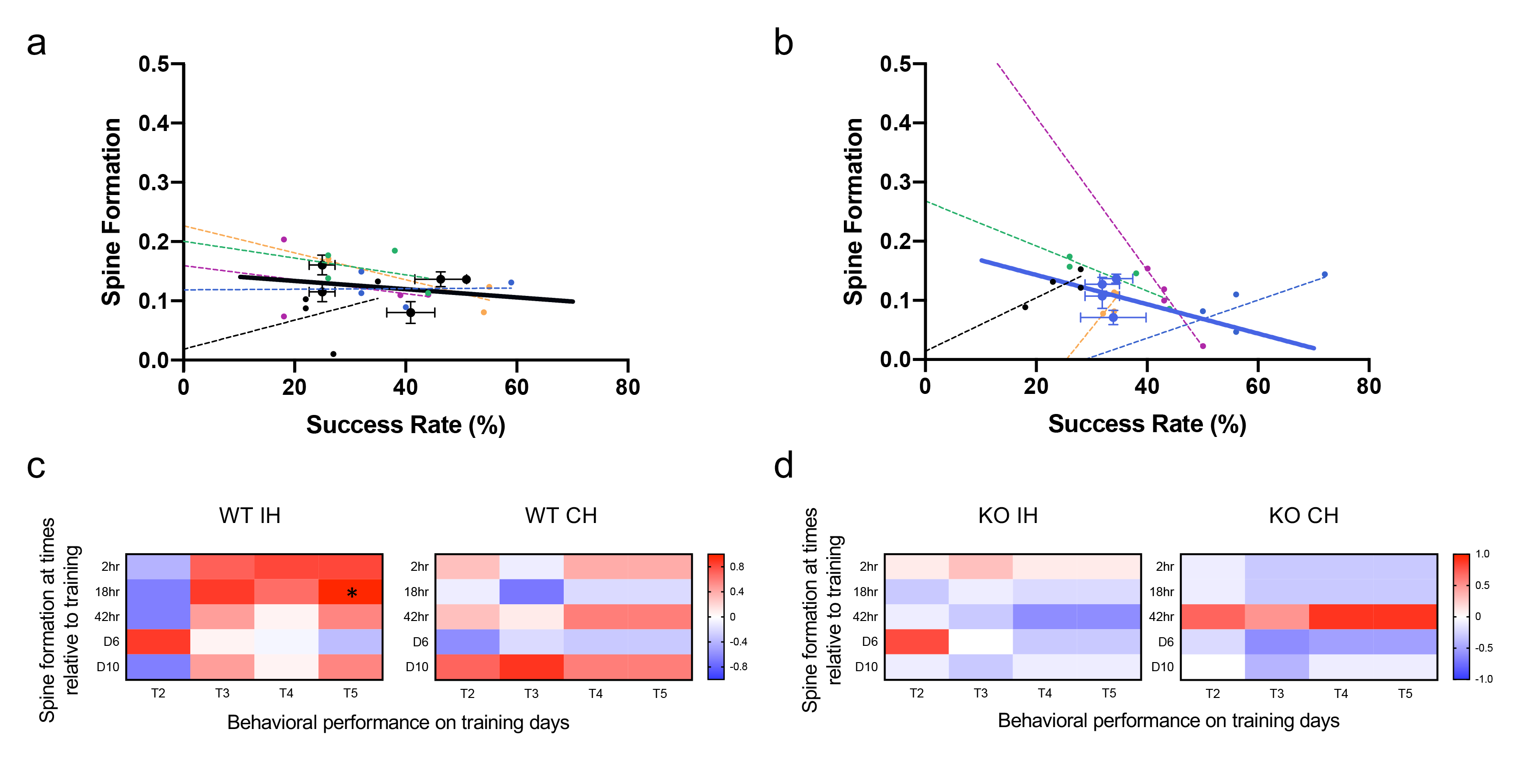

Supplement: Extended Data Figure 4-1 — Spine formation in contralateral hemisphere of WT mice does not correlate with increased behavioral performance. a, Correlation of reaching performance and spine formation at individual training sessions for which imaging was performed 2–24 h thereafter. Small symbols represent individual mice, and dashed lines are linear regressions for each mouse. Bold blue symbols represent average spine formation and behavioral performance of n = 5 mice at each imaging session for WT and Fmr1 KO (b) mice. Bold line is the linear regression for the average values WT (R2 = 0.07, p = 0.74; KO, R2 = 0.01, p = 0.88). Error bars, SEM. Correlations (Spearman coefficient) between spine formation at different time points and behavioral performance on different days in (c) WT and (d) KO mice. Download Figure 4-1, TIF file. [file enu-eN-NWR-0364-22-s10.tif]
