# Supplementary material for: Impaired AMPARs Translocation into Dendritic Spines with Motor Skill Learning in the Fragile X Mouse Model
Source: eNeuro. 2023 Mar 24;10(3):ENEURO.0364-22.2023. doi: 10.1523/ENEURO.0364-22.2023 (PMC10056836; doi:10.1523/ENEURO.0364-22.2023)
Supplement: Extended Data Table 1-1 — Full statistical information for Figure 1. Download Table 1-1, DOCX file. [file enu-eN-NWR-0364-22-s11.docx]

| Figure | Number of samples | Analysis | F statistics | P Values |
| --- | --- | --- | --- | --- |
| Fig. 1c  (Success rate Normalized) | WT n=6 mice KO n=7 mice | Repeated Measure Two-way ANOVA Posthoc One Way ANOVA with Bonferroni correction | Genotype F(1, 11)=22.68, p=0.0006  Time F(4, 44)=13.25, p<0.0001  Genotype*Time F(4, 44)=7.031, p=0.0002 | **WT**  D1 vs D2 p=0.015  D1 vs D3 p=0.001  D1 vs D4 p=0.0002  D1 vs D5 p<0.0001  **KO**  D1 vs D2 p=0.9  D1 vs D3 p=0.97  D1 vs D4 p>0.99  D1 vs D5 p=0.86  **WT vs KO**  D2 p=0.0002  D3 p=0.015  D4 p=0.0002  D5 p=0.0003 |
| Fig 1-1a  (Success rate raw data) | WT n=6 mice KO n=7 mice | Repeated Measure Two-way ANOVA Posthoc One Way ANOVA with Bonferroni correction | Genotype F(1, 11)=4.29, p=0.062  Time F(4, 44) = 12.26, p<0.0001  Genotype*Time F(4, 44) = 6.07, p=0.0006 | **WT**  D1 vs D2 p=0.16  D1 vs D3 p=0.033  D1 vs D4 p=0.008  D1 vs D5 p<0.0009  **KO**  D1 vs D2 p=0.99  D1 vs D3 p=0.99  D1 vs D4 p>0.98  D1 vs D5 p=0.86  **WT vs KO**  D2 p=0.12  D3 p=0.87  D4 p=0.08  D5 p=0.17 |
| Fig 1-1b  (Reaching attempts) | WT n=6 mice KO n=7 mice | Repeated Measure Two-way ANOVA Posthoc One Way ANOVA with Bonferroni correction | Genotype F(1, 11)=4.571, p=0.056  Time F(2.452, 26.97)=0.57, p=0.0002  Genotype * Time F(4, 44)=1.916, p=0.12 | **WT vs KO**  D1 p=0.97  D2 p=0.19  D3 p=0.77  D4 p=0.08  D5 p=0.47 |
