# Supplementary material for: Impaired AMPARs Translocation into Dendritic Spines with Motor Skill Learning in the Fragile X Mouse Model
Source: eNeuro. 2023 Mar 24;10(3):ENEURO.0364-22.2023. doi: 10.1523/ENEURO.0364-22.2023 (PMC10056836; doi:10.1523/ENEURO.0364-22.2023)
Supplement: Extended Data Table 2-1 — Full statistical information for Figure 2. Download Table 2-1, DOCX file. [file enu-eN-NWR-0364-22-s12.docx]

| Figure | Number of samples | Analysis | F statistics | P Values |
| --- | --- | --- | --- | --- |
| Fig. 2b  (total spines) | WT n=5 mice  KO n=5 mice | Nested random effects mixed model analysis. Tests for fixed effects | Genotype F(1, 9.5)=0.72, p=0.42,  Hemisphere F(1, 61.41)=21.25, p<0.0001,  Time F(4, 564.9)=12.99, p<0.0001 Genotype* Hemisphere F(1,61.41)=0.73, p=0.39  Genotype*Time F(4,564.9)=0.39, p=0.81, Condition*Time F(4,564.9)=0.87, p=0.48 Genotype*Hemisphere*Time F(4,564.9)=1.13, p=0.34 | **Ti vs T0:**  **WT IH** 2hr p=0.3, 18hr p=0.23, 42hr p=0.72, 6D p=0.33, 10D p=0.37.  **WT CH** 2hr p<0.001, 18hr p<0.001, 42hr <0.001, 6D p=0.004, 10D p=0.18  **KO IC** 2hr p=0.47, 18hr p=0.61, 42hr p=0.62, 6D p=0.93, 10D p=0.049.  **KO CH** 2hr p=0.011, 18hr p<0.001, 42hr p= 0.091, 6D p= 0.053, 10D p=0.26.    **IC vs CH:**  **WT** 2hr p=0.052, 18hr p=0.004, 42hr p=0.022, 6D p=0.006, 10D p=0.28  KO 2hr p=0.25, 18hr p=0.009, 42hr p=0.72, 6D p=0.23, 10D p=0.017  **WT vs KO:**  **IH** 2hr p=1, 18hr p=0.98, 42hr p=1, 6D p=0.94, 10D p=0.83  **CH** 2hr p=0.69, 18hr p=0.61, 42hr p=0.31, 6D p=0.83, 10D p=1 |
| Fig. 2c (Formation) | WT n=5 mice,  KO n=5 mice. | Nested random effects mixed model analysis. Tests for fixed effects | Formation:  Genotype F(1, 17.58)=0.05, p=0.83, Hemisphere F(1, 86.84)=6.43, p=0.013  Time F(4, 560)=12.54, p<0.001  Genotype*Hemisphere F(1, 86.84)=6.98, p<0.01  Genotype*Time F(4, 560)=1.45, p=0.22 Hemisphere*Time, F(4, 560) =5.77,p<0.001 Genotype*Hemisphere*Time F(4, 560) =0.29, p=0.88 | **WT CH vs IH** 2hr p<0.001, 18hr p=0.014, 42hr p=0.95, 6D p=0.07, 10D p=0.77  **KO CH vs IH** 2hr p=0.38, 18hr p=0.87, 42hr p=0.36, 6D=0.97, 10D=0.99  **WT vs KO**  **IH** 2hr p=1, 18hr p=0.98, 42hr p=0.54, 6D p=0.14, 10D p=0.197  **CH** 2hr p=0.077, 18hr p=0.62, 42hr p=0.89, 6D p=0.95, 10D p=0.9 |
| Fig. 2c (Elimination) | WT n=5 mice,  KO n=5 mice. | Nested random effects mixed model analysis. Tests for fixed effects | Elimination:  Genotype F(1, 11.36)=0.06, p=0.8  Hemisphere F(1, 100.3)=4.67, p=0.033  Time F(4, 563.2)=44.43, p<0.0001 Genotype*Hemisphere F(1, 100.3)=6.06, p=0.0155  Genotype*Time F(4, 563.2)=0.58, p=0.678 Hemisphere*Time F(4, 563.2)=0.46, p=0.764; Genotype*Hemisphere*Time F(4, 563.2)=2.19, p=0.069. | **WT CH vs IH**: 2hr p=0.85, 18hr p=0.29, 42hr p=0.61, 6D=0.17, 10D p<0.001,  **KO CH vs IH**: 2hr p=1.00, 18hr p=1.00, 42hr p=0.81, 6D p=1, 10D p=0.74.  **WT vs KO**  **IH** 2hr p=0.8, 18hr p=0.96, 42hr p=1, 6D p=1, 10D p=0.19.  **CH** 2hr p=1, 18hr p=0.64, 42hr p=1, 6D p=0.4, 10D p=0.033 |
| Fig. 2d  (New Spines stabilized) | WT n=5 mice,  KO n=5 mice. | Two-way ANOVA with post-hoc Sidak Correction | Genotype F(1, 16)=0.32, p=0.58  Hemisphere F(1, 16)=9.39 p=0.007  Hemisphere*Genotype F(1, 16)=0.15, p=0.7 | **IH vs CH**  WT p=0.053  KO p=0.14 |
| Fig. 2-1  (Spine densities at T0) | WT n=10 hemisphere,  KO n=10 hemispheres. | Two-tailed t-test |  | **WT vs KO**  P=0.0013 |
