# Supplementary material for: Impaired AMPARs Translocation into Dendritic Spines with Motor Skill Learning in the Fragile X Mouse Model
Source: eNeuro. 2023 Mar 24;10(3):ENEURO.0364-22.2023. doi: 10.1523/ENEURO.0364-22.2023 (PMC10056836; doi:10.1523/ENEURO.0364-22.2023)
Supplement: Extended Data Table 3-1 — Full statistical information for Figure 3. Download Table 3-1, DOCX file. [file enu-eN-NWR-0364-22-s13.docx]

| Figure | Number of samples | Analysis | F statistics | P Values |
| --- | --- | --- | --- | --- |
| Fig. 3b  (sGluA2 changes) | WT n=5 mice,  KO n=5 mice. | Nested random effects mixed model analysis. Tests for fixed effects | Genotype F(1,11941)=0.34, p=0.56, Hemisphere F(1,11941)=2.01, p=0.16, Genotype*Hemisphere F(1,11941)= 9.5, p=0.002,  Time F(4,11941)=55.08, p<0.001, Genotype*Time F(4,11941)= 2.38, p=0.049,  Hemisphere*Time F(4,11941)=8.06 P<0.001, Genotype*Hemisphere*tr F(4,11941)=5.38, p=0.003 | **Ti/T0:**  **WT-IH** 2hr p=0.47, 18hr p= 0.74, 42hr p= 0.43, 6D p= 0.003, 10D p<0.001,  **WT-IH** 2hr p=0.84, 18hr p=0.002, 42hr p=0.58, 6D p=1.00, 10D p=0.98.  **KO-CH** 2hr p=0.20, 18hr p= 0.004, 42hr p=0.72, 6D p = 0.20, 10D p=0.38, **KO-IH** 2hr p=1.00, 18hr p=0.61, 42hr p=1.00, 6D p=0.27, 10D p=0.092  **CH vs IH: WT** 2hr p=0.97, 18hr p<0.001, 42hr p=0.14, 6D=0.009, 10D p<0.001,  **CH vs IH KO** 2hr p=0.22, 18hr p=0.12, 42hr p=0.69, 6D=1.00,10D =0.85  **WT vs KO: CH** 2hr p=0.11, 18hr p=0.02, 42hr p=0.31, 6D=0.48, 10D p=0.03 |
| Fig. 3c | WT-Utr: N = 36 dendrites,  WT-Tr: N = 37 dendrites,  KO-Tr: N = 36 dendrites,  KO-Utr: N = 35 dendrites. | Three-Way ANOVA with posthoc one-way ANOVA with Sidak’s correction | SAME:  Genotype: F(1, 700)=1.942, p=0.16  Hemisphere: F(1, 700)=0.55, p=0.46  Time: F(4, 700) =20.71, p<0.0001  All interactions P>0.1  DOWN  Genotype: F(4, 700)=6.52, p=0.011  Hemisphere: F(4, 700)=.79, p=0.029  Time: F(4, 700)=3.512, p=0.014  Genotype*Hemisphere: F(1, 699)=5.45,  p = 0.02  All other interactions p>0.09  UP  Genotype F(1, 700)=1.401, p=0.24  Hemisphere F(1, 700)=1.39, p=0.24  Time F(4, 700)=22.55, p<0.0001  Genotype*Hemisphere: F(1, 700)=11.42, p< 0.001  All other interactions P>0.2 | DOWN  WT 18hr IH vs CH: p =0.005  WT D10 IH vs CH: p=0.072  UP  WT D10 IH vs CH: p=0.013 |
| Fig. 3e | WT-Utr: N = 36 dendrites,  WT-Tr: N = 37 dendrites,  dendrites,  KO-Tr: N = 36 dendrites,  KO-Utr: N = 35 dendrites. | Two-Way ANOVA with posthoc one-way ANOVA with Sidak’s correction | Genotype F(1, 139)=1.48, p=0.22  Genotype*Hemisphere F(1, 139)=5.283, p=0.023 | WT CH vs. WT IH: p=0.018 |
| Fig. 3-1  (Raw GluA2 intensity in basal condition) | WT-Utr: N= 880 spines,  WT-Tr: N= 956 spines,  KO-Tr:N=  966 spines,  KO-Utr:N= 910 spines | Two-sample Kolmogorov-Smirnov test |  | WT-Tr vs KO-Tr:  D = 0.10954, p-value = 1.965e-05  WT-Utr vs KO-Utr:  D = 0.096603, p-value = 0.0004728 |
| Fig. 3-2  (dendrite intensity) | WT n=5 mice,  KO n=5 mice. | Nested random effects mixed model analysis. Tests for fixed effects | Genotype F(1,12042)=0.08, p=0.7  Hemisphere F(1,12042)=17.04, p<0.0001  Time F(4,12042)=21.54, p<0.0001  Genotype*Hemisphere F(1,12042)=1.61, P=0.2, Genotype*Time F(4,12042)=19.47, p<0.0001, Hemisphere*Time F(4,12042)=6.46, p<0.0001, Genotype* Hemisphere*Time F(4,12042)=14.67,p<0.0001 | Ti/T0: WT-CH, p>0.5 for all time points  Ti/T0: WT-IH, p>0.5  Ti/T0: KO -CH, T42 hr p=0.002, 10D p=0.033, rest p>0.05  Ti/T0: KO -IH, 2hr p=0.04, 6D p=0.22, 10D p=0.009, rest p>0.05 |
| Fig. 3-4  (spine intensity) | WT n=5 mice,  KO n=5 mice. | Nested random effects mixed model analysis. Tests for fixed effects | Genotype F(1,11941)=0.88, p=0.34, Hemisphere F(1,11941)=1.41, p=0.23, Genotype*Hemisphere F(1,11941)= 3.64, P=0.056, Time F(4,11941)=14.98, p<0.001, Genotype*Time F(4,11941)= 0.77, p=0.55, Hemisphere*Time F(4,11941)=1.54 p=0.18, Genotype*Hemisphere*Time F(4,11941)=0.83, p=0.51 | Ti/T0: WT-CH 2hr p=0.8, 18hr p= 0.47, 42hr p= 0.45, 6D p=0.75, 10D p=0.73,  WT-IH 2hr p=0.072, 18hr p=0.017, 42hr p=0.031, 6D p<0.001, 10D p<0.001.  KO- CH2hr p=0.023, 18hr p=0.009, 42hr p=0.021, 6D p<0.001, 10D p<0.001  KO-IH 2hr p=0.37, 18hr p=0.2, 42hr p=0.006, 6D p=0.001, 10D p<0.001  CHvs IH: WT 2hr p=0.51, 18hr p=0.44, 42hr p=0.59, 6D=0.033, 10D p=0.046,  CH vs IH KO 2hr p=0.64, 18hr p=0.67, 42hr p=0.99, 6D p=1.00,10D p=1.00. |
| Fig. 3-5 | WT-IH: N = 36 dendrites,  WT-CH: N = 37 dendrites,  WT-untrained mice: N=25 dendrites,  KO-CH: N = 36 dendrites,  KO-IH N = 35 dendrites. | Ordinary one-way ANOVA with Sidak’s correction | Hemisphere: F (2, 94) = 4.602, p=0.0124 | WT CH vs. WT IH: p= 0.0137  WT CH vs. WT No Hemisphere: p=0.04 |
| Fig.3-6  (Four groups) | WT n=5 mice,  KO n=5 mice | Nested random effects ANOVA model. | Genotype F(1,11948)=0.46,p=0.4975;  Hemisphere F(1,11948)=8.32, p=0.0039; Genotype*Hemisphere F(1,11948)=10.69, p=0.0011;  Time F(4,11948) =59.22, p<.00001; Genotype*Time F(4,11948)=2.75,p=0.0266;  Hemisphere*Time F(4,11948)=8.58,p<0.0001;Groups F(3,11948) =94.78,p<0.0001; Genotype*Group F(3,11948)=2.40,p=0.0658;Hemisphere*Group F(3,11948)=5.00,p=0.0018,Time*Group F(12,11948)=2.83,p=0.0007; Genotype*Hemisphere*Time F(4,11948)=5.67, p=0.0001, Genotype*Hemisphere*Group F(3,11948)=5.81, p= 0.0006. | Ti/T0: Group 1: WT-CH: T 2hr, 18hr,42hr,6D,10D, p<0.001.  Ti/T0: Group 1: WT-IH: 2hr p=0.19, 18hr p=0.63, 42hr p=0.57, 6D,10D p<0.001.  Group comparision at 10D:  WT- CH: grp 1 VS 2, p<0.001  WT- CH: grp 1 VS 3, p<0.001  WT- CH grp 1 VS 4, p<0.001  WT- CH; grp 2 VS 3, p=0.066  WT- CH: grp 2 VS 4, p=0.042  WT- CH: grp 3 VS 4, p=1  WT-IH: grp 1 VS 2 p<0.001  WT-IH: grp 1 VS 3 p<0.001  WT-IH: grp 1 VS 4 p<0.001  WT-IH: grp 2 VS 3 p=0.015  WT-IH: grp 2 VS 4 p =0.014  WT-IH: grp 3 VS 4 p = 1.00  Ti/T0: Group 1: KO-CH: T 2hr p=0.38, 18hr p=0.6, 42hr p=0.012, 6D p=0.006, 10D p<0.001.  Ti/T0: Group 1: KO-IH: T 2hr p=0.92, 18hr p=0.35, 42hr p=0.13, 6D p=0.002, 10D p<0.001  Group comparision at 10D:  KO- CH: grp 1 VS 2, p<0.001  KO- CH: grp 1 VS 3, p<0.001  KO CH: grp 1 VS 4, p<0.001  KO- CH; grp 2 VS 3, p=0.95  KO- CH: grp 2 VS 4, p=0.92  KO- CH: grp 3 VS 4, p=0.55  KO-IH: grp 1 VS 2 p<0.051  KO-IH: grp 1 VS 3 p<0.001  KO-IH: grp 1 VS 4 p<0.001  KO-IH: grp 2 VS 3 p<0.001  KO-IH: grp 2 VS 4 p <0.001  KO-IH: grp 3 VS 4 p = 1.00 |
